# Hierarchical Organization of Biomaterials in the Formation of Photonic Nanostructures

**DOI:** 10.1002/advs.78639

**Published:** 2026-11-10

**Authors:** Sangyeop Kim, Jihun Kang, Seunghwan Moon, Liliana D'Alba, Matthew D. Shawkey, Jong‐Souk Yeo

**Affiliations:** ^1^ School of Integrated Technology College of Computing Yonsei University Incheon Republic of Korea; ^2^ BK21 Graduate Program in Intelligent Semiconductor Technology Yonsei University Seoul Republic of Korea; ^3^ Evolution and Optics of Nanostructures Group Department of Biology Ghent University Ghent Belgium; ^4^ Naturalis Biodiversity Center Leiden CR The Netherlands

**Keywords:** biomaterials, hierarchical organization, nanostructures, self‐assembly, structural colors

## Abstract

Biological organisms produce diverse nanostructures, some of which generate structural color through their interaction with light. The distinctive properties of structural color have inspired efforts across multiple fields to reproduce these effects through bottom‐up approaches using natural and synthetic materials. Despite substantial research on the optical mechanisms of structural coloration, how natural photonic nanostructures develop, particularly how molecular interactions and biological regulation enable higher‐order organization, remains incompletely understood, despite their potential to inspire new low‐cost methods of manufacturing nanostructures. This review provides a comprehensive overview of organization into photonic nanostructures, focusing on four of the most prevalent biomaterials (cellulose, chitin, corneous beta‐proteins (CBPs), and melanin). We first examine their formation using a common hierarchical framework encompassing: 1) biological regulation and synthesis, 2) molecular building blocks, 3) intermediate structural units, 4) mesoscale organization, and 5) photonic architecture and optical function. We then discuss the molecular interactions and mesoscale assembly mechanisms that connect these hierarchical levels, together with computational approaches ranging from atomistic and molecular simulations to continuum modeling and artificial intelligence. By integrating biological, physical, and computational perspectives, this review clarifies how natural photonic nanostructures develop and provides a foundation for biomimetic approaches to nanostructural fabrication.

## Graphical abstract

Natural organisms create vibrant structural colors through precisely organized nanostructures formed under mild biological conditions. This review explores how cellulose, chitin, corneous beta‐protein, and melanin form complex nanostructures across molecular, mesoscale, and tissue levels. By integrating biological, physical, and computational perspectives, it establishes a framework for understanding and engineering hierarchical photonic nanostructures.

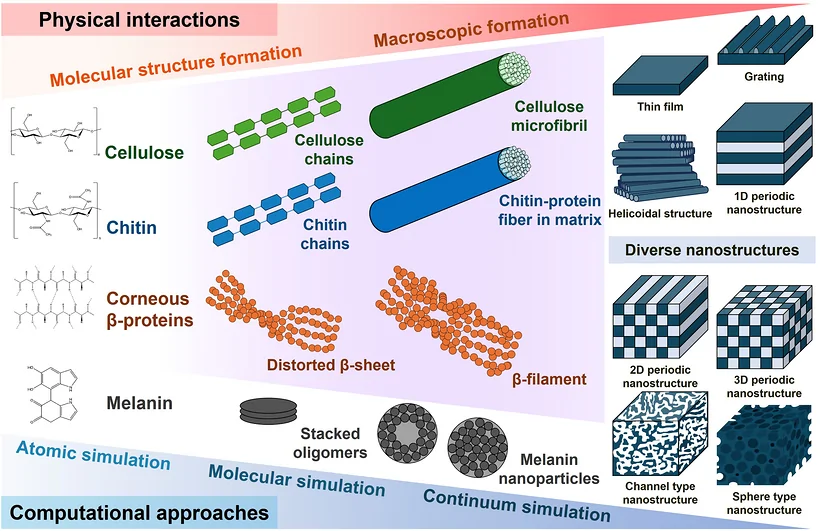

## 1 Introduction

Optically active nanostructures produce structural coloration by manipulating light to generate a wide range of iridescent and non‐iridescent colors without relying solely on wavelength‐selective absorption by pigments. Over millions of years of evolution, organisms have developed structural colors that remain vivid under sunlight, are resistant to harsh environmental conditions, and are formed under biological parameters, including neutral pH (6.5–8.0), physiological temperatures (37–40°C), and ambient pressure (1 atm) [1, 2, 3, 4]. Although the optical mechanisms of many natural structural colors have been extensively characterized, the processes by which their underlying nanostructures form remain much less understood [5, 6]. A central challenge is that photonic structures are formed through processes spanning multiple size scales, as molecular composition and interactions are translated into ordered or quasi‐ordered structures at subcellular and tissue levels.

In particular, understanding sub‐nanometer effects is critical, as chemical structures of core biomaterials largely determine their assembly into larger‐scale structures [7, 8, 9, 10, 11, 12, 13, 14, 15, 16, 17, 18]. Nevertheless, how this chemistry governs assembly into tissue‐level structure remains poorly understood. To address this gap, we organize our discussion around a common hierarchical framework consisting of five conceptual levels: 1) biological regulation and synthesis, 2) molecular building blocks, 3) intermediate structural units (e.g., microfibrils, filaments, or oligomeric aggregates formed by the assembly of molecular building blocks), 4) mesoscale organization, and 5) photonic architecture and optical function. Figure 1 provides a schematic overview of this hierarchy. We focus on four materials commonly used in nature to produce structural colors: cellulose [19], chitin [20], corneous beta‐proteins(CBPs) [21], and melanin [22, 23]. Each material‐specific section first provides a brief photonic context, followed by discussion according to these five hierarchical levels.

**FIGURE 1:**
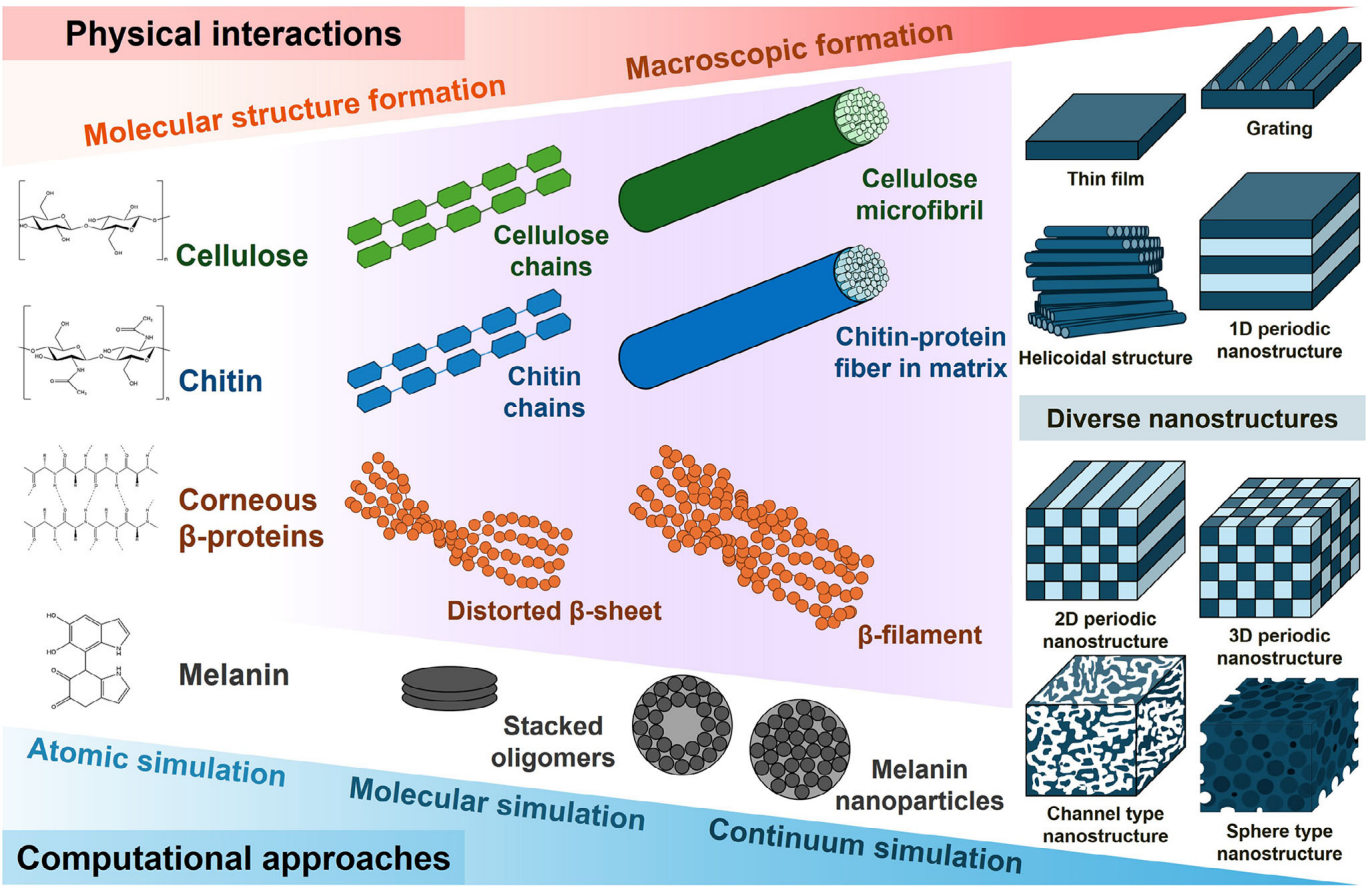
Schematic representation of the hierarchical structure of biomaterials, illustrating physical interactions and computational approaches.

Throughout this review, we reserve the term “architecture” for this final, fully assembled level, using “structure” or “nanostructure” elsewhere. For clarity, we use “biological assembly” to describe organization that is regulated by biological components, whereas “self‐assembly” refers to processes driven primarily by intrinsic physicochemical interactions.

This review primarily examines how biological regulation, physical interactions, and computational approaches together drive the formation of natural photonic nanostructures across these hierarchical levels. Because these processes are often difficult to observe directly, we place particular emphasis on the physical interactions that connect molecular‐ and mesoscale organization before reviewing computational approaches that, together with new empirical data, could further advance understanding. Accordingly, this review is intended for two groups of readers: materials, photonics, and nanoscience researchers seeking to reproduce natural photonic nanostructures by emulating the principles governing their formation rather than only their final geometries; and biologists seeking physical and computational approaches to test proposed mechanisms of nanostructure formation.

## 2 Biomaterials

This section reviews current knowledge of four major biomaterials that form natural photonic nanostructures: cellulose, chitin, corneous beta‐proteins (CBPs), and melanin. Although other biomaterials, including guanine [24] and collagen [25, 26], also contribute to structural coloration, these four are emphasized because they provide contrasting yet comparable examples of how molecular biosynthesis, nanoscale organization, and higher‐order assembly are coupled in the formation of photonic structures. By comparing these materials within a common hierarchical framework, we aim to identify shared organizational principles that may explain their recurrent use in natural nanostructures.

### 2.1 Cellulose

#### 2.1.1 Photonic Context

Cellulose, the most abundant naturally occurring polysaccharide [27, 28, 29, 30], is a major component of several plant nanostructures that generate structural color [31]. Foundational investigations of structural coloration in plant leaves date to the 1970s, when blue iridescence in tropical understory plants such as *Selaginella* was explored by Lee and Lowry [32]. Subsequent ultrastructural studies related this coloration to thin‐film and lamellar structures [33]. Later comparative work indicated that the ultrastructural basis of blue leaf iridescence can differ between species, involving multilayered cellulose microfibrils in epidermal cell walls and lamellar structures within specialized plastids [34]. These early studies revealed the structural basis of plant iridescence and offered essential context for later investigations of cellulose‐based photonic structures. More recent studies show that iridescent blue observed in certain fruits, including *Pollia condensata* and *Margaritaria nobilis*, results from helicoidal multilayers of cellulose that interfere with light (Figure 2A) [19, 35], enabling reflection of circularly polarized light across a range of wavelengths [19, 36]. Detailed overviews of various natural photonic structures and optical mechanisms are available in other reviews [37, 38].

**FIGURE 2:**
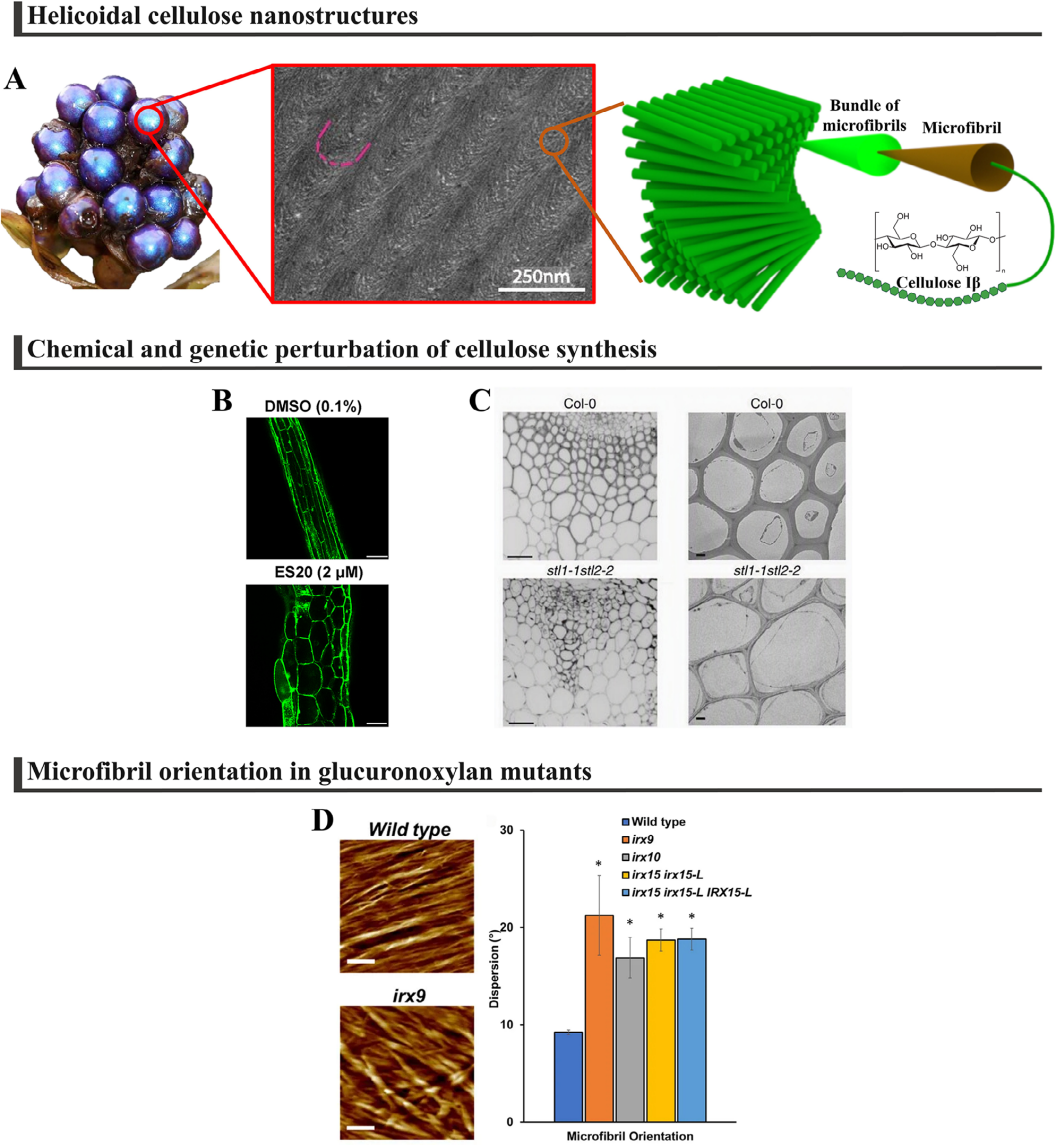
Cellulose nanostructures in nature. (A) Vivid structural coloration in *Pollia condensata* resulting from its helicoidal nanostructures composed of cellulose microfibrils [19]. Adapted with permission from ref [19], Copyright 2012, National Academy of Sciences. (B) Inhibition of CESA catalytic activity by ES20, leading to altered growth of *Arabidopsis* compared to the control (DMSO). Scale bars, 100 µm [39]. Adapted under the terms of the CC BY 4.0 license from ref [39], Copyright 2020, The Authors, published by Oxford University Press on behalf of the American Society of Plant Biologists. (C) Comparative analysis of stem cross‐sections (left) and cell walls (right) in *Arabidopsis* between control (Col‐0) and STL double mutant (stl1‐1 stl2‐2). The stl1‐1 stl2‐2 mutant exhibits thinner cell walls than Col‐0. Scale bars: 50 µm (left) and 2 µm (right) [40]. Adapted under the terms of the CC BY 4.0 license from ref [40], Copyright 2016, The Authors, published by Springer Nature. (D) (Left) Atomic force microscopy images displaying cellulose fibril orientation in wild‐type (wt) and glucuronoxylan‐deficient mutants (irx9) of *Arabidopsis*. Scale bars, 100 nm. (Right) Cellulose microfibril orientations in wt and glucuronoxylan mutants (irx9, irx10, irx15 irx15‐L, and irx15 irx15‐L IRX15‐L). Glucuronoxylan mutants exhibit increasingly disorganized cellulose fibril arrays relative to wt [41]. Adapted under the terms of the CC BY 4.0 license from ref [41], Copyright 2021, The Authors, published by Frontiers. Lowercase gene symbols (e.g., irx15‐L) denote loss‐of‐function mutant alleles, whereas uppercase symbols (e.g., IRX15‐L in the irx15 irx15‐L IRX15‐L line) denote a wild‐type IRX15‐L transgene reintroduced as a complementation control, included to account for ecotype background differences between the irx15 and irx15‐L T‐DNA lines. **Abbreviations**: CESA, cellulose synthase; ES20, Endosidin20; DMSO, dimethyl sulfoxide; wt, wild‐type; irx9, irregular xylem 9; irx10, irregular xylem 10; irx15, irregular xylem 15; IRX15‐L, irregular xylem 15‐like; STL, STELLO proteins.

#### 2.1.2 Biological Regulation and Synthesis

In plants, cellulose is primarily synthesized via cellulose synthase (CESA) complexes (CSCs). CESA, a glycosyltransferase family 2 (GT2) protein, produces β‐1,4‐glucan from the substrate uridine diphosphate (UDP)‐α‐D‐glucose and is embedded in the plasma membrane [42]. CESA is synthesized in the endoplasmic reticulum and transported to the Golgi apparatus for CSC assembly. The CSC exhibits a rosette‐shaped structure consisting of six subunits, although the exact number of CESAs within a rosette remains debated: earlier models proposed six CESAs per subunit (36 total), whereas later structural and computational studies supported three CESAs per subunit (18 total), corresponding to a debated range of 18 to 36 CESAs per rosette [43, 44, 45]. CSC activity is influenced by multiple factors, which in turn determine the quantity and morphology of synthesized cellulose chains. For example, Liu et al. [39] report that Endosidin20 inhibits CESA catalytic activity, resulting in the synthesis of shortened, non‐close‐packed cellulose chains (Figure 2B). Zhang et al. [40] demonstrate that Golgi‐localized STELLO (STL) proteins interact with CESA to regulate CSC assembly. STL mutants exhibit altered CSC spatial distribution, producing a significantly thinner secondary cell wall (SCW) than that of the controls (Figure 2C). Sophisticated helicoidal nanostructures that interact with light to produce color are prominently located in the SCW [46]. Therefore, CSC activity, which determines the total thickness of the photonic structure and that of the secondary wall, is a key factor in shaping cellulose nanostructures. Following β‐1,4‐glucan chain synthesis involving 18–36 CESAs, individual chains assemble into cellulose microfibrils.

#### 2.1.3 Molecular Building Blocks

Cellulose exists in four distinct forms: cellulose I, II, III, and IV. This review focuses on cellulose I, the only naturally occurring form [47, 48, 49, 50]. Cellulose I is further divided into two subtypes: cellulose Iα, predominantly found in bacteria and algae, and cellulose Iβ, abundant in higher plants [47]. Cellulose consists of linear chains of D‐anhydroglucopyranose units linked via β‐1,4‐glucosidic bonds [51]. Each glucose unit is rotated 180° relative to its neighbors, facilitating intra‐ and intermolecular hydrogen bonds (H‐bonds) that stabilize individual cellulose chains.

#### 2.1.4 Intermediate Structural Units

These interactions promote aggregation of linear chains into microfibrils with high tensile strength [52, 53, 54]. Microfibrils contain amorphous and crystalline regions. Cellulose from bacteria or algae consists entirely of crystalline regions, while higher plant cellulose exhibits lower crystallinity and a greater proportion of amorphous regions [55, 56, 57, 58, 59]. In higher plants, microfibril diameters are typically ∼ 3 nm [60, 61, 62]. Chains are connected through H‐bonds; however, the precise mechanism of self‐assembly remains unclear. Assembly may occur spontaneously via interchain H‐bonds [63], or proteins, such as chitinase‐like proteins, may affect chain aggregation [64].

#### 2.1.5 Mesoscale Organization

These microfibrils align in parallel arrays, forming multilayers with slight rotational offsets, known as helicoidal structures [65, 66, 67]. The layers are periodically spaced, producing specific colors through Bragg reflection from the helicoidal multilayers [19, 35]. After assembly, microfibrils form a relatively thin primary cell wall (PCW), followed by the formation of a thick, rigid SCW between the PCW and plasma membrane. Cellulose in the PCW has a relatively low degree of polymerization, short chain length, and contains xyloglucan, while cellulose in the SCW has a higher degree of polymerization, longer chains, and contains xylan. Consequently, the mechanical properties of the two cell walls differ [68]. Cellulose microfibrils in the SCW align through interactions with other cell components, including hemicellulose and lignin, to form periodic structures. Busse‐Wicher et al. [69] demonstrate that specific acetylation patterns of xylan, a hemicellulose, contribute to the formation of helicoidal SCW structures by interacting with cellulose microfibrils. Furthermore, Crowe et al. report the effect of xylan (glucuronoxylan) on SCW structures by comparing wild‐type (wt) and xylan mutants [41], showing that xylan modulates microfibril assembly by influencing their coalescence (Figure 2D). Conversely, lignin acts as a natural adhesive, linking cellulose and hemicellulose. Kang et al. demonstrate that lignin predominantly binds to xylan rather than directly to cellulose and hemicellulose in SCW, with xylan mediating the cellulose‐lignin interaction [70].

#### 2.1.6 Photonic Architecture and Optical Function

The development of cellulose nanostructures is governed by two fundamental processes: glucan chain synthesis by CESAs and subsequent assembly mediated by interactions with other cell wall components, including hemicellulose and lignin. CESA composition and regulation, together with interactions among cellulose microfibrils, hemicellulose, and lignin, determine cellulose nanostructure morphology by modulating the precise arrangement and periodicity of microfibrils [39, 41, 43, 44, 45, 69, 70, 71, 72, 73, 74]. These multifaceted interactions and regulatory processes contribute to the formation of helicoidal cellulose multilayers with distinctive optical properties. However, the biological mechanisms linking cellulose synthesis to the mesoscale organization of microfibrils into helicoidal architecture, and ultimately, structural color remain incompletely understood, particularly how variation in CESA/CSC activity and cell wall composition is translated into the pitch and long‐range order of helicoidal multilayers.

### 2.2 Chitin

#### 2.2.1 Photonic Context

Chitin, the second most abundant polysaccharide in nature after cellulose, forms the exoskeletons of arthropods and other organisms [20, 75, 76]. It produces brilliant structural colors by forming nano‐ and microscale cuticle structures, as observed in the iridescent wings of butterflies (Figure 3A) [77]. Butterfly wings contain chitin‐based photonic crystals within their scales, often forming complex 3‐dimensional (3D) structures, such as gyroids or tree‐like architectures (Figure 3A,B). These structures selectively reflect specific wavelengths of light, producing vivid colors [78, 79, 80, 81]. Similar to cellulose photonic structures, chitin‐based structures in certain beetles function as multilayer reflectors [82, 83]. Some exhibit helicoidal arrangements of chitin macromolecules analogous to cellulose microfibrils (Figure 3A,B), generating colors through the pitch of helicoidal nanostructures [84, 85].

**FIGURE 3:**
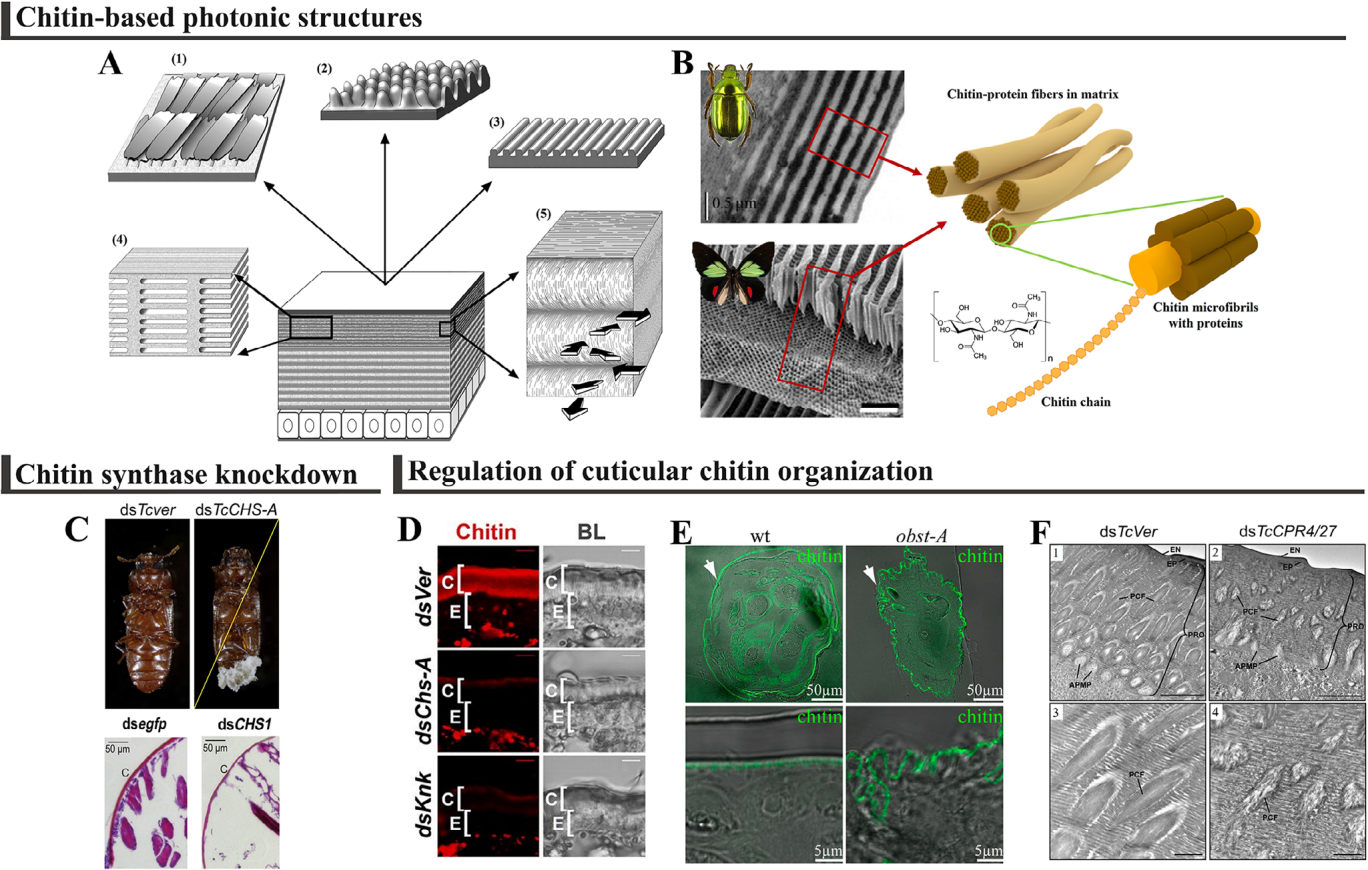
Chitin nanostructures in nature. (A) Classification of chitin photonic structures categorized by type and location. Scales (1) and photonic cuticle surfaces (2–3) located in the epicuticle, primarily found in butterflies and moths, while multilayer thin films (4) and helicoidal arrays (5) occur in the exocuticle, predominantly found in beetles [77]. Adapted with permission from ref [77], Copyright 2009, Elsevier Inc. (B) Electron microscopy images of nanostructures composed of chitin chains and proteins in *Gastrophysa viridula* [86] and *Parides childrenae* [79]. Scale bars, 0.5 µm (top) and 1 µm (bottom). *Gastrophysa viridula* image: Adapted with permission from ref [86], Copyright 2013, AIP Publishing. *Parides childrenae* image: Adapted under the terms of the CC BY license from ref [79], Copyright 2014, The Authors, published by BioMed Central. (C) (Top) Effect of dsRNA treatment targeting TcCHS‐A on the cuticle formation of *Tribolium castaneum*, compared with ds*TcVer*‐treated control. TcCHS‐A knockdown results in incomplete cuticle layer development [87]. Adapted with permission from ref [87], Copyright 2008, Elsevier Ltd. (Bottom) Hematoxylin‐eosin‐stained head cuticle images of *Henosepilachna vigintioctopunctata* following oral RNAi‐mediated HvCHS1 knockdown. HvCHS1‐knockdown larvae exhibit a thinner and incompletely developed cuticle compared with the control treated with double‐stranded RNA targeting enhanced green fluorescent protein [88]. Adapted with permission from ref [88], Copyright 2021, Elsevier Inc. (D) Chitin distribution in *Tribolium castaneum* following RNAi‐mediated knockdown of CHS1 (dsChs‐A) and Knk (dsKnk), compared with the dsVer‐treated control. (C, cuticle; E, epithelial cells) Chitin staining and bright‐field images (BL) show altered cuticular chitin organization after knockdown of either gene [89]. Adapted with permission from ref [89], Copyright 2011, National Academy of Sciences. (E) Chitin‐stained larval cross‐sections of wild‐type (wt) and *obst‐A* null mutant *Drosophila*. *obst‐A* mutants exhibit a wrinkled epidermal cuticle and disrupted cuticle integrity compared with the wild type larvae [90]. Adapted under the terms of the CC BY license from ref [90], Copyright 2015, The Authors, published by the American Society for Biochemistry and Molecular Biology. (F) Cross‐sectional TEM images of elytra from *Tribolium castaneum* following RNAi‐mediated depletion of TcCPR4 and TcCPR27 (dsTcCPR4/27), compared with the ds*TcVer*‐treated control. TcCPR4/27 depletion results in disorganized horizontal laminae and abnormal vertical pore canals containing amorphous fibrous material [91]. Adapted under the terms of the CC BY license from ref [91], Copyright 2015, The Authors, published by PLOS. Key structural features are labelled: EN, EP, PRO, PCF, APMP. Scale bars: 2 µm (panels 1, 2) and 500 nm (panels 3, 4). **Abbreviations**: dsRNA, double‐stranded RNA; TcCHS‐A, CHS1 gene in *Tribolium castaneum*; dsTcVer, control dsRNA in *Tribolium castaneum* (used in Arakane et al. [87] and Noh et al. [91]); dsVer, control dsRNA targeting the *vermilion* gene in *Tribolium castaneum* (used in Chaudhari et al. [89]); RNAi, RNA interference; HvCHS1, chitin synthase 1 gene in *Henosepilachna vigintioctopunctata*; CHS1, chitin synthase 1; Knk, Knickkopf; wt, wild‐type; *obst‐A*, *obstructor A* mutant; TEM, transmission electron microscopy; CPR, cuticular protein containing the Rebers and Riddiford Consensus; dsTcCPR4/27, dsRNA‐mediated knockdown of CPR genes in *T. castaneum*; EN, envelope; EP, epicuticle; PRO, procuticle; PCF, pore canal fiber; APMP, apical plasma membrane protrusion.

#### 2.2.2 Biological Regulation and Synthesis

Chitin is synthesized through the polymerization of UDP‐N‐acetylglucosamine (UDP‐GlcNAc) catalyzed by chitin synthase (CHS), a member of the glycosyltransferase family 2 (GT2) [92, 93]. In insects, two major CHS genes exist, CHS1 and CHS2 [94]. CHS1 is primarily expressed in the cuticle and is essential for exoskeletal chitin development, while CHS2 is predominantly expressed in gut epithelial cells and produces chitin associated with the peritrophic membrane [95]. RNA interference (RNAi) uses double‐stranded RNA (dsRNA) to promote degradation of a target mRNA, thereby reducing gene expression [96, 97]. This reduction in gene expression is referred to as gene knockdown, whereas gene knockout involves genetic disruption that abolishes gene function; both approaches enable gene function to be investigated through the resulting phenotype [98]. Arakane et al. [87, 99] identified two CHS genes in *Tribolium castaneum*, TcCHS1 and TcCHS2 (also referred to as TcCHS‐A and TcCHS‐B, respectively), and demonstrated through RNAi experiments in whole insects that TcCHS1 is primarily involved in cuticular chitin formation, whereas TcCHS2 is required for the synthesis of peritrophic membrane‐associated chitin in the midgut (Figure 3C). Similarly, Jiang et al. [88] demonstrated through oral RNAi experiments in whole larvae that depletion of HvCHS1 impairs cuticle formation in *Henosepilachna vigintioctopunctata* (Figure 3C). Ingestion of dsRNA targeting HvCHS1 reduced cuticle thickness and delayed formation of the newly generated cuticle layer during ecdysis. These findings support an essential role for CHS1 in cuticular chitin formation and provide a biological basis for understanding the development of chitin‐based nanostructures.

#### 2.2.3 Molecular Building Blocks

Chitin (β‐(1→4)‐poly‐N‐acetyl‐D‐glucosamine) is the primary structural material in marine organisms, fungi, and insects, analogous to cellulose in plants [100]. Chitin chains are assembled from GlcNAc monomers linked via β‐1,4‐glycosidic bonds. Each monomer carries an acetamido groups at C‐2, which enhances intra‐ and intermolecular H‐bonding between neighboring chains [101, 102]. It occurs in three forms, α, β, and γ, which determine microfibril orientation.

#### 2.2.4 Intermediate Structural Units

The most common, α‐chitin, exhibits an antiparallel molecular arrangement and is stabilized by numerous intra‐ and intermolecular H‐bonds during microfibril formation [103]. These chitin microfibrils can exceed 0.5 µm in length and are often assembled into bundles, containing ≥ 10 parallel‐aligned microfibrils [12]. Conversely, β‐ and γ‐chitin have fewer H‐bonds, resulting in higher hydration and reduced compactness of chitin chains within microfibrils. Consequently, α‐chitin, predominantly found in arthropod exoskeletons, forms rigid structures that support stable structural coloration, but β‐ and γ‐chitin are typically associated with flexible structures where structural coloration is rare [12, 104, 105]. Thus, structural diversity in chitin‐based organisms, from nano‐ to macroscopic scales, arises from the three chitin forms, each with distinct microfibril orientations. Structures responsible for coloration, such as butterfly wings and insect exoskeletons, are predominantly composed of α‐chitin owing to its superior crystalline stability [106, 107, 108]. A comprehensive review of chitin chemistry and photonic structures is provided by Jiaxin Hou [20]. The synthesized chitin chains are extruded into the extracellular space, where they self‐assemble into chitin microfibrils and nanostructures through hydrogen bonding (H‐bonding), Van der Waals forces (vdW), hydrophobic interactions, and electrostatic attractions [20, 94, 109].

#### 2.2.5 Mesoscale Organization

In multilayer structures of *Coleoptera*, synthesized chitin chains aggregate into fibrils that assemble into bundles integrated with specific structural cuticular proteins (CPs), discussed below [109]. These bundles are subsequently aligned in parallel to form layers that are precisely laminated into helicoidal multilayers similar to those of cellulose [109, 110]. During development, chitin structures in butterfly scales are formed in association with an actin cytoskeleton, which serves as a template [111]. CPs, such as the Knickkopf (Knk) family, obstructor proteins, and cuticular proteins containing the Rebers and Riddiford Consensus (CPR), are crucial for chitin assembly. For instance, RNAi experiments in whole *Tribolium castaneum* insects demonstrated that CHS1 is required for cuticular chitin formation, whereas Knk is involved in the organization and protection of newly synthesized chitin from chitinase‐mediated degradation (Figure 3D) [89]. Additionally, obstructor proteins regulate Serpentine and Knk [112]. In *Drosophila*, *obst*‐A null mutants exhibit disrupted extracellular matrix organization and a wrinkled, disorganized chitin structure (Figure 3E) [90]. Furthermore, Noh et al. [113] report that CPR27 and CPR18 in the rigid cuticle of the red flour beetle, *Tribolium castaneum*, determine cuticle layer morphology and rigidity, as CPR depletion results in structurally disorganized cuticle layers. Similarly, RNAi experiments in whole *Tribolium castaneum* insects showed that TcCPR4 and TcCPR27 contribute to the organization of horizontal laminae and chitin‐containing pore canals in the elytral cuticle, as their simultaneous depletion produces disorganized laminae and abnormal pore canal morphology (Figure 3F) [91]. Thus, the formation of higher‐order chitin architectures following synthesis via CHS depends on the spontaneous self‐assembly of chitin chains through H‐bonds and coordinated interactions with diverse cuticular constituents. These variants can differ in the arrangement, diameter, length, and crystallinity of chitin microfibrils and nanostructures, which are key determinants of structural coloration and light interaction.

#### 2.2.6 Photonic Architecture and Optical Function

Chitin nanostructures responsible for structural coloration in insects exhibit diverse morphologies across species, including multilayers in beetle exocuticles and surface gratings on butterfly exocuticles. In butterfly wing scales, α‐chitin is the primary component, and larger photonic architectures, including photonic crystals, gyroids, and tree‐like structures, have been studied [78, 114, 115, 116]. These structures consist primarily of a chitin‐air nanocomposite with a periodic chitin array at the exocuticle surface [117]. Uniquely, *Cyphochilus* and *Lepidiota stigma* produce ultra‐white structural coloration through highly interconnected, disordered α‐chitin networks [118]. Chitin nanostructure formation is therefore governed collectively by CHS‐mediated chain synthesis, physicochemical interactions that promote higher‐order chitin organization, and CP‐mediated biological assembly [111, 119, 120]. Further elucidation of CHS catalytic and transport mechanisms remains important for understanding how molecular‐scale synthesis is translated into chitin‐based photonic nanostructures.

### 2.3 Corneous Beta‐proteins

#### 2.3.1 Photonic Context

Corneous beta‐proteins (CBPs) were historically referred to as β‐keratins because of their β‐sheet‐rich structure and characteristic X‐ray diffraction pattern in avian and reptilian corneous tissues [121]. However, subsequent molecular and structural studies showed that these proteins are distinct from keratins, which belong to the intermediate filament protein family, leading to the adoption of the term corneous beta‐proteins [122]. In this review, we therefore use the term CBPs and focus specifically on their role as a major structural component of avian feather photonic nanostructures. Structural coloration of birds is primarily observed in the barbs and barbules of feathers, with rare exceptions in structures such as beaks [123] and skin [25]. In feathers, CBPs organize into photonic nanostructures, such as channel‐ and sphere‐type nanostructures, determined by their short‐range order (Figure 4A) [124, 125, 126, 127, 128, 129, 130, 131]. A comprehensive overview of the feather hierarchical structure is provided in this review [132]. Because avian feather nanostructures predominantly contain CBPs, understanding the biological factors that regulate CBP organization is crucial for elucidating their developmental mechanisms [133]. This review therefore focuses on CBPs as the principal structural component of mature avian feather photonic nanostructures. However, in some species, such as the rock dove (*Columba livia*), structural color arises from thin‐film interference in the CBP cortex, while melanin enhances spectral contrast by absorbing transmitted light and suppressing multiple internal reflections [134]. CBPs are involved in several structurally distinct components of feather coloration, including CBP‐based nanostructures and the CBP cortex of barbules, which can contribute to thin‐film interference. Their organization therefore appears to vary among different feather structures and mechanisms of structural coloration.

**FIGURE 4:**
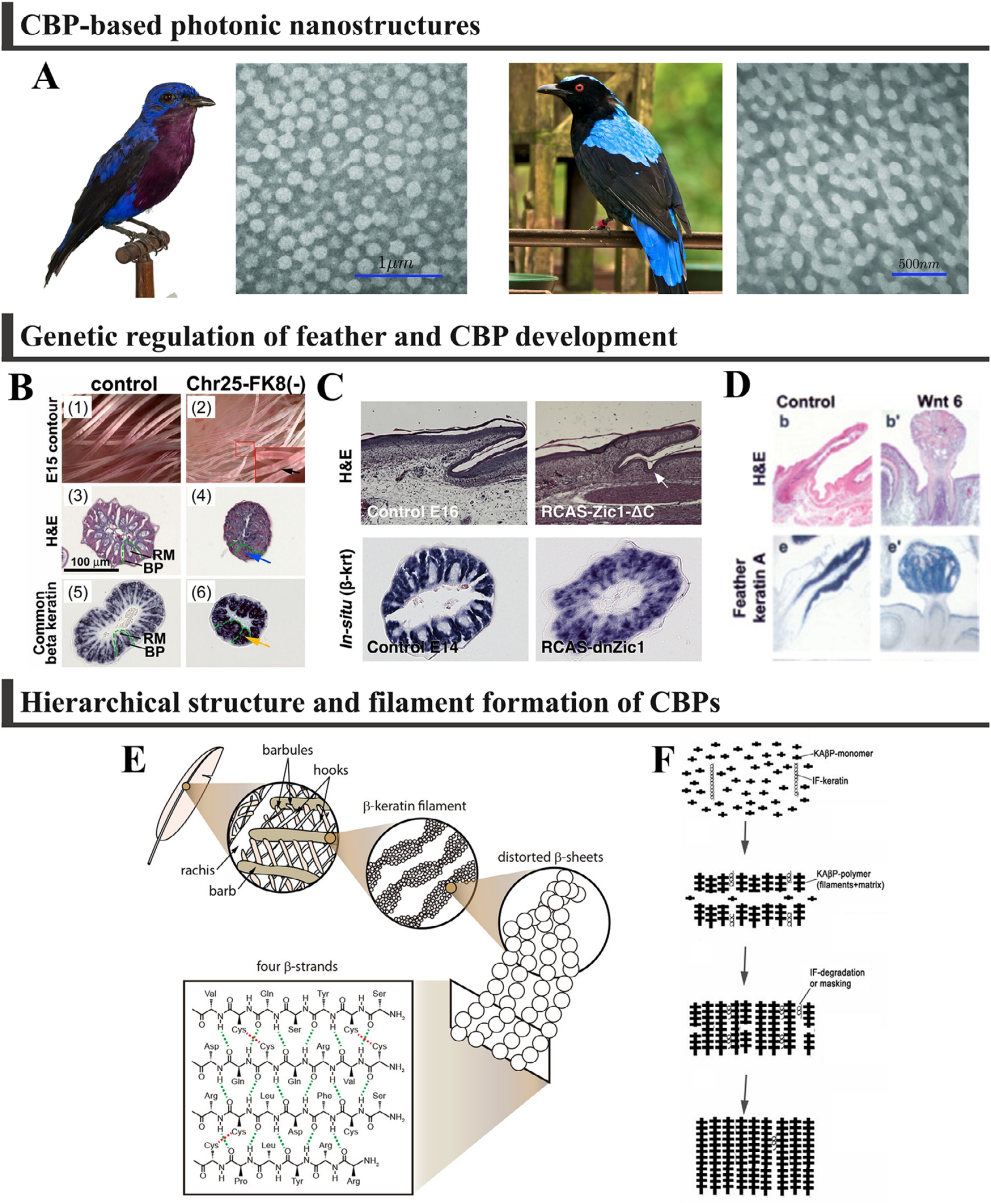
CBP nanostructures in nature. (A) (left) *Cotinga cotinga* and its sphere‐type CBP nanostructure observed through TEM. (right) *Irena puella* and its channel‐type CBP nanostructure captured using TEM [135]. Digital images: C. cotinga, copyright Übersee‐Museum Bremen, CC BY‐SA 4.0; I. puella, copyright Bob Owen, CC BY 2.0. TEM images adapted with permission from ref [135], Copyright 2010, Wiley‐VCH Verlag GmbH & Co. KGaA, Weinheim. (B) Comparison of control and Chr25‐FK8(‐) (suppressed feather CBP 8 on chromosome 25). (1–2) Bright‐field images showing thinner and twisted (black arrow) feather filaments in Chr25‐FK8(‐) than those of the control. (3–4) H&E‐stained images showing abnormal barb ridges and fewer medullary cells (blue arrow) in Chr25‐FK8(‐). (5–6) In situ hybridization using common CBP probes revealing smaller CBP‐negative domains (yellow arrow) [136]. Adapted with permission from ref [136], Copyright 2015, National Academy of Sciences. (C) Comparative analysis of control, constitutively active Zic1 (RCAS‐Zic1‐ΔC), and dominant‐negative Zic1 (RCAS‐dnZic1). (Top) RCAS‐Zic1‐ΔC shows invagination in H&E images. (Bottom) In situ hybridization showing disrupted CBP expression in RCAS‐dnZic1 [137]. Adapted with permission from ref [137], Copyright 2020, Elsevier Inc. (D) Effect of Wnt6 on feather buds and CBP. (Top) Wnt6 mutant with enlarged feather buds compared to that of the control in cross‐sectional view of H&E‐stained images. (Bottom) Irregular feather CBP arrays in Wnt6 mutant [138]. Adapted with permission from ref [138], Copyright 2003, Society for Investigative Dermatology, Inc. (E) Hierarchical structure of avian feathers composed of CBPs [139]. Adapted with permission from ref [139], Copyright 2018, American Chemical Society. (F) Formation of dense CBP (KaβP) filaments accompanied by degradation or masking of α‐keratin (IF) in avian feathers [140]. Adapted with permission from ref [140], Copyright 2012, Elsevier GmbH. **Abbreviations**: BP, barb plate; RM, ramus; CBP, corneous beta‐protein; TEM, transmission electron microscopy; KaβP, keratin‐associated beta‐protein; IF, intermediate filament; RCAS, replication‐competent avian sarcoma virus; H&E, hematoxylin and eosin; E, embryonic day.

#### 2.3.2 Biological Regulation and Synthesis

Feather CBPs are synthesized from keratinocytes in epidermal appendages through transcription and translation of DNA and RNA and are encoded by multiple genes in tandem arrays on specific chromosomes. Chromosome numbers vary among avian species [141], but Greenwold et al. [142] report that, across 48 avian genomes, most feather CBP genes are located on chromosomes 1, 2, 6, 7, 10, 25, and 27. Wu et al. [136] examined the effects of feather keratin gene (FK) expression on feather and CBP morphogenesis in developing chicken embryos. Feathers with suppressed FK8 on chromosome 25 show reduced medullary cell number, irregular feather filaments, and abnormal cell morphology compared to those of the control (Figure 4B). Moreover, transcription factors (TFs) regulating morphogenesis‐related genes also exert significant effects. Thus, the influence of genes across different chromosomes and the expression of feather CBPs under multiple TFs contribute to the generation of diverse avian feather structures [143, 144]. Bhattacharjee et al. analyzed the genomes of chicken (*Gallus gallus domesticus*) and turkey (*Meleagris gallopavo*), identifying numerous TFs that regulate feather CBP genes [144]. Liang et al. [137] report additional TFs influencing CBP morphogenesis; for instance, the zinc finger protein of the cerebellum 1 (Zic1), a TF regulating feather CBP genes on chromosome 27 in chickens, induces abnormal feather filament morphology (Figure 4C). Non‐TF proteins also contribute to feather and CBP development. Chodankar et al.  [138] report that misexpression of Wnt6, a member of the Wnt family involved in feather follicle development, using a replication‐competent avian sarcoma virus (RCAS) in developing chicken embryos resulted in enlarged feather buds and disorganized feather CBP (Figure 4D). These findings indicate that feather CBP development is influenced both by direct CBP transcription and indirect mechanisms, such as feather cell signaling pathways.

#### 2.3.3 Molecular Building Blocks

Corneous proteins form the structural backbone of diverse animals and are composed of long amino acid chains, such as glycine, cysteine, and serine [145]. Keratins and CBPs are present in developing avian feathers, and most photonic structures in mature feathers consist of CBPs with β‐sheet structures (Figure 4E) [124, 142, 146]. In keratins, amino acid chains form right‐handed α‐helices stabilized by intermolecular H‐bonds [147]. However, in CBPs, the central region of amino acid sequences participates in β‐sheet formation [14]. While CBPs contain amino acids similar to those in α‐keratin, they are enriched in serine, glycine, and proline, resulting in structural variation [148]. Furthermore, a lower cysteine concentration potentially results in reduced mechanical properties owing to fewer disulfide bonds, whereas a higher concentration can enhance mechanical strength [149]. Additionally, the sequences responsible for β‐sheet formation and the surrounding matrix exhibit considerable variation, resulting in differences in β‐sheets and matrix concentrations, alongside the sizes of assembled structures. Following transcription, individual polypeptide chains of feather CBPs are synthesized through translation. These chains adopt a twisted conformation, forming a distorted β‐sheet structure ∼2 nm in diameter. Fraser and Parry [14] characterized the number and roles of amino acids in these chains by analyzing avian and reptilian CBP sequences using X‐ray diffraction (XRD). Feather CBPs consist of ∼100 amino acids, with an average of 32 residues forming the filament and the remaining regions constituting the matrix. Amino acid composition is consistent across species, with specific sites showing identical amino acid types. For instance, prolines at folded sites facilitate β‐sheet formation [150]. Additionally, conserved amino acids potentially mediate strong interactions during chain assembly. However, beyond these 32 conserved residues, the number and types of amino acids constituting the matrix vary significantly, influencing the formation of secondary and higher‐order feather CBP structures [151, 152] along with their interactions with light. Furthermore, because amino acid composition determines the solubility parameter [153] of feather CBPs, a factor identified by Prum et al.  [16] as crucial for photonic nanostructure formation under phase‐separation theory, such variations can lead to distinct CBP nanostructure morphologies.

#### 2.3.4 Intermediate Structural Units

Following single‐chain formation, CBP dimers are formed through the assembly of two β‐sheets with a diameter of 2–5 nm and longitudinal pitches of 7–10 nm [154, 155]. Subsequently, CBP dimers assemble with keratin‐associated proteins and other keratins to form filament‐matrix structures [121, 156]. The filament comprises CBPs filaments alongside keratin intermediate filaments (α‐keratin), whereas the matrix consists primarily of terminal domains of CBPs filaments and α‐keratin [157]. In contrast to claws, scales, and beaks, intermediate filaments in feathers are eliminated during later stages of development through degradation or masking, leaving CBP filaments as the dominant structural component in mature feathers (Figure 4F) [140]. However, the chicken frizzle feather phenotype is potentially formed by α‐keratin mutations, indicating that intermediate filaments are vital for feather development [158]. Considering these results, although intermediate filaments are absent in mature feathers, interactions between CBPs and other proteins are crucial for CBP filament assembly during development.

#### 2.3.5 Mesoscale Organization

CBP‐rich material within feather barb medullary cells is proposed to self‐assemble through phase separation and subsequent kinetic arrest, giving rise to channel‐ and sphere‐type nanostructures [16, 159]. Electron microscopic observations in this system reveal no membrane‐ or organelle‐based scaffold, such as a cytoskeletal or endoplasmic‐reticulum template, that could prepattern the nanostructure; rather, medullary cells appear to act by setting the initial physicochemical parameters, including CBP concentration and drying, that subsequently govern self‐assembly through phase separation and its arrest.

#### 2.3.6 Photonic Architecture and Optical Function

CBP nanostructures in blue and some green feather barbs produce bright and highly saturated colors. These structures are generally described as quasi‐ordered, as they exhibit neither highly long‐range periodic order and nor complete disorder. Several decades ago, non‐iridescent blue coloration in feathers was often attributed to incoherent scattering from randomly distributed nanostructures [160]. Subsequent studies, however, using Fourier analysis of electron micrographs and small‐angle X‐ray scattering (SAXS), demonstrated that these quasi‐ordered CBP structures are isotropic yet possess a characteristic spatial periodicity on a length scale comparable to visible wavelengths [124, 161]. These findings provided strong evidence that the observed coloration arises predominantly from coherent scattering associated with short‐range structural order rather than from purely incoherent scattering. Underlying this optical tunability, CBP nanostructures are formed through these complex biological processes, from specific gene transcription, regulated by numerous TFs, to CBP assembly. The amino acid sequences within the filament and matrix regions are crucial for determining the final CBP structures. However, comprehensive studies show that the fundamental morphologies of CBP nanostructures may be partially predetermined during gene transcription, which governs amino acid sequence composition. Therefore, further research is required to elucidate how interspecific variations in amino acid sequences influence the formation of CBP‐based photonic nanostructures. Such studies will advance understanding of the developmental mechanisms underlying these structures in nature.

### 2.4 Melanin

#### 2.4.1 Photonic Context

Melanin is a naturally occurring polymeric pigment present across diverse organisms, including bacteria, fungi, plants, and animals [162, 163, 164]. Melanin, with its high refractive index (1.8–2.0) and broad absorption spanning ultraviolet to visible light, is crucial in generating non‐iridescent feather coloration in birds through broadband absorption [22, 165, 166, 167]. Conversely, ordered crystalline arrangement of melanosomes, embedded within materials such as CBP or air, produces iridescent structural colors [21, 168, 169, 170, 171]. The wing feathers of green‐winged teal (*Anas crecca*) and wild turkey (*M. gallopavo*) contain solid and hollow melanosome rods, respectively, contributing to their bright structural coloration (Figure 5A). Additionally, melanosomes display diverse morphologies, including spherical, rod‐shaped, and platelet forms, and can be organized into single or multilayered arrays [172, 173, 174, 175, 176]. Moreover, melanosome‐based structural coloration can be modulated by the overlying CBP cortex, whose thickness influences the resulting iridescent color [177, 178, 179].

**FIGURE 5:**
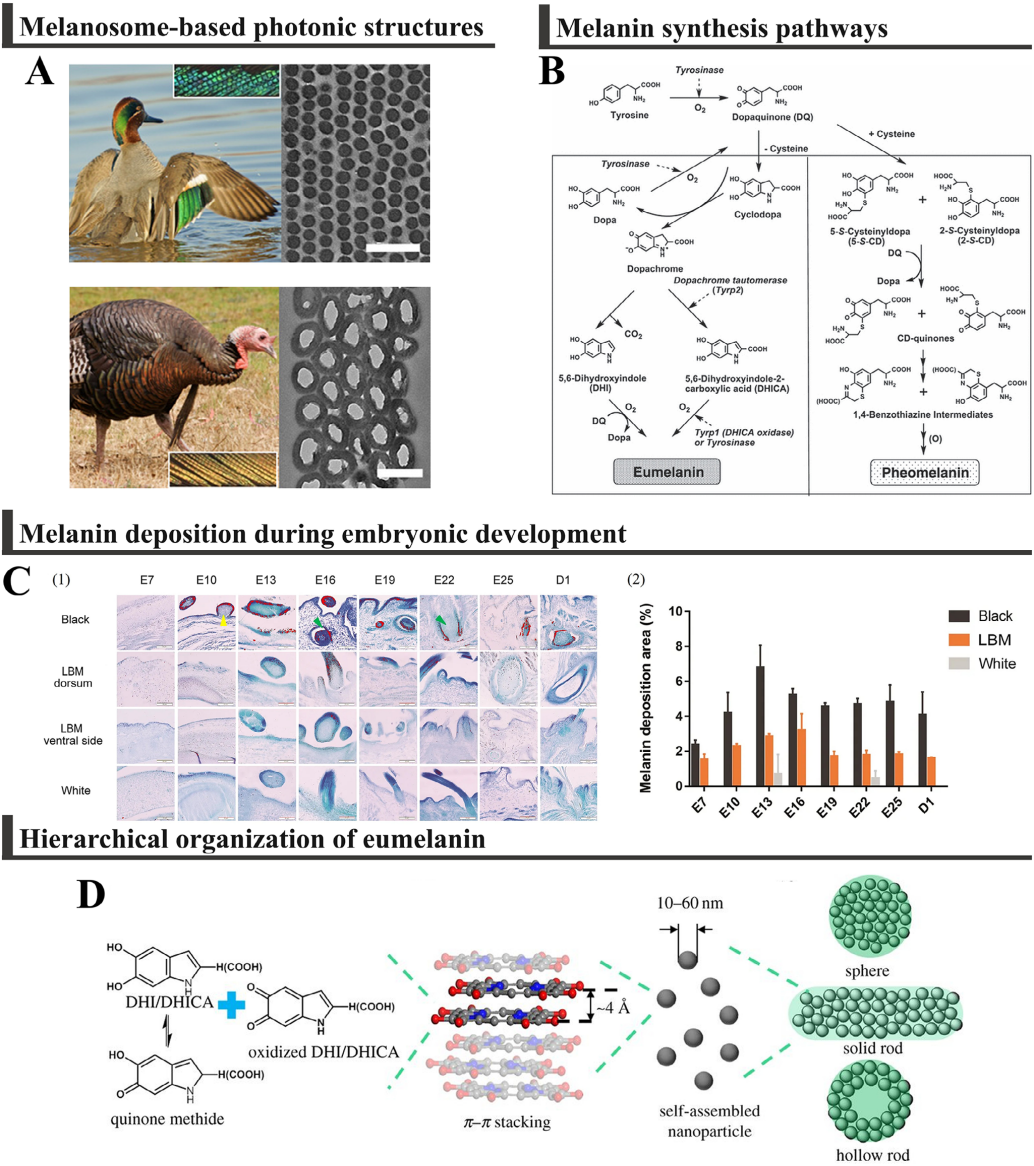
Melanin nanostructures in nature. (A) Representative biological examples illustrating melanosome‐based structural coloration (scale bar: 500 nm) [174]. Adapted under the terms of the CC BY‐NC 4.0 license from ref [174], Copyright 2017, The Authors, exclusive licensee American Association for the Advancement of Science. (B) Biogenic pathways of eumelanin and pheomelanin synthesis [180]. Adapted with permission from ref [180], Copyright 2007, John Wiley & Sons. (C) Developmental dynamics of melanin deposition. (1) Spatial distribution of melanin deposition in embryos of three breeds from embryonic day 7–25 (E7–25) and on day 1 post‐hatching (D1). Red particles indicate visible melanin; yellow and green arrowheads indicate feather buds and feather follicles, respectively. (2) Melanin deposition area (%) quantified across developmental stages for Black, LBM, and White ducks [181]. Adapted under the terms of the CC BY 4.0 license from ref [181], Copyright 2020, The Authors. (D) Hierarchical organization of natural eumelanin, from eumelanin monomers and π–π‐stacked molecular assemblies to subunit nanoparticles and eumelanosomes with distinct morphologies [182]. Adapted with permission from ref [182], Copyright 2018, The Royal Society. **Abbreviations**: CC, Creative Commons; E, embryonic day; D, day after hatching; LBM, light brown mottling.

#### 2.4.2 Biological Regulation and Synthesis

Melanin can be classified into five types: eumelanin, pheomelanin, and neuromelanin in animals, and pyomelanin and allomelanin primarily in microorganisms and plants [165]. This review focuses on the two predominant types of animal melanin, black‐to‐brown eumelanin and yellow‐to‐reddish pheomelanin. Despite their distinct chemical compositions and optical properties, both originate from a common precursor (Figure 5B), DOPAquinone (DQ), which is generated through tyrosinase‐catalyzed oxidation of L‐tyrosine or DOPA to form DOPAchrome [180, 183, 184].

Notably, melanosome biogenesis constitutes a second, distinct layer of biological regulation beyond the enzymatic synthesis of melanin precursors. During eumelanosome development, proteolytically processed PMEL (Premelanosome protein) forms amyloid‐like fibrils that assemble into a matrix supporting subsequent melanin polymerization, condensation, and storage [164, 185]. Melanosomes develop through four morphologically defined stages in melanocytes, categorized into two main phases: an unpigmented phase involving PMEL fibril formation (stages I and II) and a pigmented phase involving melanin synthesis (stages III and IV) [186, 187]. In stage I, the nascent eumelanosome is a membrane‐bound vesicle devoid of pigments and is characterized by the presence of the melanocyte protein PMEL17 and initial intralumenal protein fibrils [188]. By stage II, these fibrils are fully formed, and the melanosomes adopt an ellipsoidal shape, with PMEL17 serving as a crucial structural component [189]. In pheomelanosomes, PMEL17 expression is significantly downregulated in cells producing pheomelanin, a process modulated by agouti signaling [190, 191]. This reduction in PMEL17 potentially contributes to the irregular shape of pheomelanosomes [192, 193]. During stage III, melanin synthesis is initiated with the delivery of integral membrane proteins, including tyrosinase, tyrosinase‐related protein (TYRP)‐1, and TYRP‐2, which localize to fibrils within the melanosome. In stage IV, melanin deposition fully obscures the internal melanosome structure. Ellipsoidal eumelanosomes, which contain eumelanin deposited on the fibrillar matrix, are generally larger than pheomelanosomes. Finally, stage IV melanosomes are transported from epidermal melanocytes to keratinocytes.

Avian coloration arises from diverse pigmentary and structural mechanisms [194]. While previous studies have focused on structural colors, the developmental mechanisms of nanostructures and the genetic basis of structural coloration remain largely unexplored.

Xi et al. [181] proposed molecular regulatory mechanisms underlying dorsoventral color variation in light brown mottling (LBM) ducks (Anas platyrhynchos) by identifying eight melanogenesis‐related differentially expressed genes (DEGs) through comparisons of dorsal and ventral skin during embryonic development, in which melanin deposition differed markedly among breeds and developmental stages (Figure 5C[1]). Quantification of melanin deposition area across these stages showed that melanin accumulation peaks at E13 in Black and at E16 in LBM embryos before stabilizing at a lower level, is consistently greater in Black than in LBM embryos, and remains minimal in White embryos throughout development (Figure 5C[2]). Gao et al.  [195] identified four melanogenesis‐related genes, attractin, endothelin receptor B, KIT proto‐oncogene tyrosine‐protein kinase, and agouti signaling protein, by detecting lineage‐specific variations in *Chrysolophus*. These genes may contribute to variation in eumelanin and pheomelanin production by altering the relative abundance of both pigments. Rubenstein et al*. *[196] investigated the molecular and genetic basis of feather development in superb starlings (*Lamprotornis superbus*) using functional genomics. They compared pigment‐related genes upregulated in iridescent blue and non‐iridescent red feathers. In iridescent feathers, TYRP‐1, Ras‐related protein, and sodium/potassium/calcium exchanger 5 were upregulated. In non‐iridescent feathers, upregulated genes included tyrosinase, G protein‐coupled receptor 143, major facilitator superfamily domain‐containing protein 12, and the precursor to norrin [196].

#### 2.4.3 Molecular Building Blocks

DOPAchrome is subsequently converted into 5,6‐dihydroxyindole (DHI) and 5,6‐dihydroxyindole 2‐carboxylic acid (DHICA), with tyrosinase‐related proteins TYRP‐1 and TYRP‐2 contributing to this pathway [164, 197]. DHI and DHICA represent the two major molecular building blocks of eumelanin, which is formed through their oxidative coupling together with their redox derivatives, yielding a complex and chemically heterogeneous pigment [198, 199]. In the pheomelanin pathway, redox interactions with DQ instead form thiol adducts of dopa (cysteinyldopa), which requires a sufficient concentration of L‐cysteine within the melanosome. Subsequent oxidation of these thiol compounds generates 1,4‐benzothiazine intermediates, the corresponding building blocks, which are further transformed into pheomelanin [180, 200].

#### 2.4.4 Intermediate Structural Units

Natural eumelanin has been proposed to exhibit a hierarchical organization in which DHI‐ and DHICA‐derived molecular units associate through π–π stacking to form subunit nanoparticles that are subsequently incorporated into eumelanosomes with distinct morphologies (Figure 5D) [182]. More broadly, melanin nanoparticle formation has been described as involving oligomer formation followed by π–π stacking‐driven nanoaggregation and further packing stabilized by hydrogen bonding and van der Waals interactions [201]. In animals, melanin is typically contained within submicron melanosomes derived from early endosomal membranes [164]. Iridescent feather barbules predominantly contain eumelanin‐rich melanosomes of diverse shapes, whereas pheomelanin‐rich melanosomes are generally spherical [5, 22]. Eumelanosomes comprise melanin nanoparticles measuring 30–50 nm, where melanin precursors are cross‐linked and organized through π‐π interactions [182]. Melanosomes exclusively composed of eumelanin or pheomelanin are rare, as most contain a mix of both pigments [202].

#### 2.4.5 Mesoscale Organization

Maia et al. [203] report that melanosomes self‐assemble through depletion‐attraction forces during keratinization, driving nanostructure formation. In wild turkeys, melanosomes initially form as solid bodies within melanocytes and subsequently develop a hollow core through the selective loss of a chemically less stable central component, likely pheomelanin, after their transfer to barbule cells [204]. This selective loss, together with the close‐packing of melanosomes, occurs largely after the death of the barbule cell and shows no evidence of active cytoskeletal guidance, consistent with self‐assembly rather than active cellular process (i.e., biological assembly).

#### 2.4.6 Photonic Architecture and Optical Function

The biogenesis of melanin and melanosomes, including precursor monomers, enzymes, and biosynthetic pathways, has been extensively characterized. While the physical basis of melanin‐based structural coloration is increasingly understood [205, 206, 207, 208, 209], the developmental mechanisms and genetic determinants remain largely unexplored. Recent availability of avian genomic datasets provides a promising opportunity to identify genes associated with structural color [196, 210].

### 2.5 Common Hierarchical Principles and Distinct Assembly Pathways

Table 1 summarizes the hierarchical formation pathways of the four biomaterials examined in this Review. Despite pronounced differences in chemistry, these systems can be organized within a common hierarchical framework in which biological regulation and synthesis establish molecular building blocks that give rise to intermediate structural units, followed by mesoscale organization and ultimately mature photonic architectures. This framework provides a common basis for comparing how chemically distinct biomaterials generate optical structures through both shared and material‐specific processes.

**TABLE 1:** Overview of interactions and assembly mechanisms in natural biomaterials.

Biomaterial	Biological regulation	Monomer	Interaction (between monomers)	∼ 1 nm	Interaction	∼100 nm	Mechanism	Photonic structure
Cellulose Iβ (higher plants) 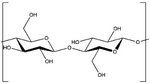	CESA, CSC	D‐glucose	β‐1,4‐glycosidic linkage	Cellulose chain	H‐bond Van der Waals forces	Cellulose microfibrils with hemicellulose and lignin	Liquid crystal self‐assembly [211]	1.Helicoidal [19]
α‐Chitin (arthropods) 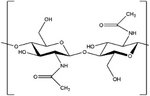	CHS1, CHS2	N‐acetyl‐glucosamine	β‐1,4‐glycosidic linkage	Chitin chain	H‐bond	Chitin‐protein fibers in mineral‐protein matrix	Liquid crystal self‐assembly Phase separation [81] Template [81]	1. Helicoidal 2. Multilayers [1] 3. Grating [1] 4. Photonic crystal [212] 5. Disordered network [118]
CBP (birds) 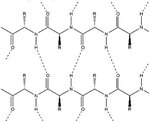	CBP genes, TFs	Amino acids (∼ 32 per 100 amino acids contribute to filament formation) [14]	Peptide bond	β‐sheet	Disulfide bond H‐bond	CBP with other proteins (α‐keratin and keratin‐associated proteins) [121, 156]	Phase separation [16]	1. Channel‐type [135] 2. Sphere‐type [135] 3. Thin film [177]
Eumelanin (birds) 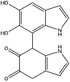	Tyrosinase, TYRP	DHI, DHICA	C–C bond (Oxidative coupling)	Oligomer	π‐π stacking H‐bond	Melanin nanoparticle within PMEL17‐scaffolded melanosome	Depletion attraction [203]	1. Single layer [172] 2. Multilayer [173]

**Abbreviations**: CBP, corneous beta‐protein; CESA, cellulose synthase; CHS1/CHS2, chitin synthase 1/2; CSC, cellulose synthase complex; DHI, 5,6‐dihydroxyindole; DHICA, 5,6‐dihydroxyindole‐2‐carboxylic acid; H‐bond, hydrogen bond; PMEL17, premelanosome protein 17; TF, transcription factor; TYRP, tyrosinase‐related protein.

The pathways linking these hierarchical levels, however, differ substantially among the four materials. Cellulose, chitin, and CBPs are polymeric systems in which molecular chains or polypeptides form fibrillar or filamentous intermediate structural units, such as cellulose microfibrils [52, 53, 54], chitin microfibrils [103], and CBP dimers [154, 155], before mesoscale organization. In contrast, melanin follows a distinct pathway in which precursor formation and oligomerization proceed within a melanosome scaffold established through PMEL‐driven biogenesis, culminating in nanoparticle formation prior to subsequent mesoscale organization [175]. Importantly, this biogenesis introduces an additional layer of biological regulation, given that the organelle serving as the intermediate structural unit is itself developmentally governed.

The balance between physicochemical self‐organization and biological direction also varies across materials and hierarchical levels. Cellulose microfibril formation and liquid‐crystalline ordering involve strong contributions from intrinsic physicochemical interactions while occurring under biologically defined cell‐wall conditions [41]. Chitin similarly exhibits physicochemical interactions during fibril formation, but its mesoscale organization additionally involves cuticular proteins and actin‐based cytoskeletal templating [111]. In CBP‐based feather nanostructures, phase separation and kinetic arrest provide a physicochemical route to mesoscale organization under biologically established molecular and cellular conditions [16]. Melanosome organization likewise combines biologically regulated organelle formation with subsequent physicochemical interactions, including depletion attraction [203]. Thus, self‐assembly and biological assembly represent overlapping contributions whose relative importance varies across hierarchical levels and material systems.

## 3 Physical Interactions

Whereas Section 2 examined the hierarchical formation of each biomaterial individually, Section 3 focuses on the underlying physical interactions and assembly mechanisms that operate across these material systems. Specifically, Section 3.1 addresses molecular‐scale interactions underlying structural assembly, whereas Section 3.2 examines mechanisms governing mesoscale organization toward complete photonic structures. Biological systems contain hierarchical nanostructures assembled from diverse chemical components [6, 38, 213, 214, 215, 216]. Understanding structure formation often involves examining the molecular interactions that govern material assembly and properties. These interactions are fundamental in nanoscience and nanotechnology, as they drive structural organization and also influence macroscopic properties [217, 218, 219, 220]. Nanostructure formation is initiated with molecular‐scale attractions and repulsions driven by noncovalent interactions. These interactions, including H‐bonds, vdW forces, and π–π interactions, are crucial for molecular assembly and structural stability [221, 222, 223]. The origins and characteristics of non‐covalent interactions differ significantly from those of covalent interactions. Non‐covalent interactions operate without orbital overlap and can act over longer ranges, from several angstroms to tens of angstroms. This is in contrast to covalent bonds, which are typically less than 2 Å [224].

At the macroscopic scale, structural colors are generated from self‐assembled nanostructures. We discuss several proposed self‐assembly mechanisms underlying nanostructure formation in natural organisms composed of the aforementioned biological materials. For instance, phase separation is crucial for nanostructure formation in arthropods, birds, and beetles [118, 159, 212]. Depletion attraction has been suggested to play a key role in melanosome organization in birds [203]. Additionally, some arthropods form through lipid‐bilayer self‐assembly [212], whereas some fruits develop their structures through liquid crystal self‐assembly [211].

### 3.1 Molecular Structure Formation for Photonic Nanostructure

#### 3.1.1 Hydrogen Bonds

Hydrogen bonds are key in determining the molecular structure and properties of biological materials. They form when a hydrogen atom covalently bonded to an electronegative donor interacts with another electronegative atom serving as the acceptor.

Cellulose I consists of a mixture of triclinic (Iα) and monoclinic (Iβ) crystalline forms (crystal symmetry of the repeating unit cells) in varying proportions [225]. Cellulose Iβ is more stable than Iα, as its abundant hydroxyl groups enable extensive intra‐ and intermolecular H‐bonding, influencing the parallel alignment of cellulose chains and their crystalline structures (Figure 6A[1]) [226, 227]. In cellulose I, intramolecular H‐bonds connect the hydroxyl group of one glucose unit to the oxygen of the next ring. These bonds extend from the O3–H hydroxyl to the O5’ ring oxygen across the glycosidic bond, and form the O2’–H to the O6 hydroxyl of the next unit [228]. Intermolecular O6–H···O3 bonds, which hold chains edge‐to‐edge in flat sheets, stabilize and organize the sheets, thereby promoting the parallel arrangement of multiple chains into elementary fibrils. Additional CH···O interactions also contribute to stability [54, 229]. Nishiyama et al. report intra‐ and intermolecular H‐bonding patterns using XRD and neutron diffraction (Figure 6A(2,3)). Cellulose Iα corresponds to a triclinic P1 unit cell with a single cellulose chain [228]. Cellulose Iβ corresponds to a monoclinic P2_1_ unit cell with two non‐identical cellulose chains [54].

**FIGURE 6:**
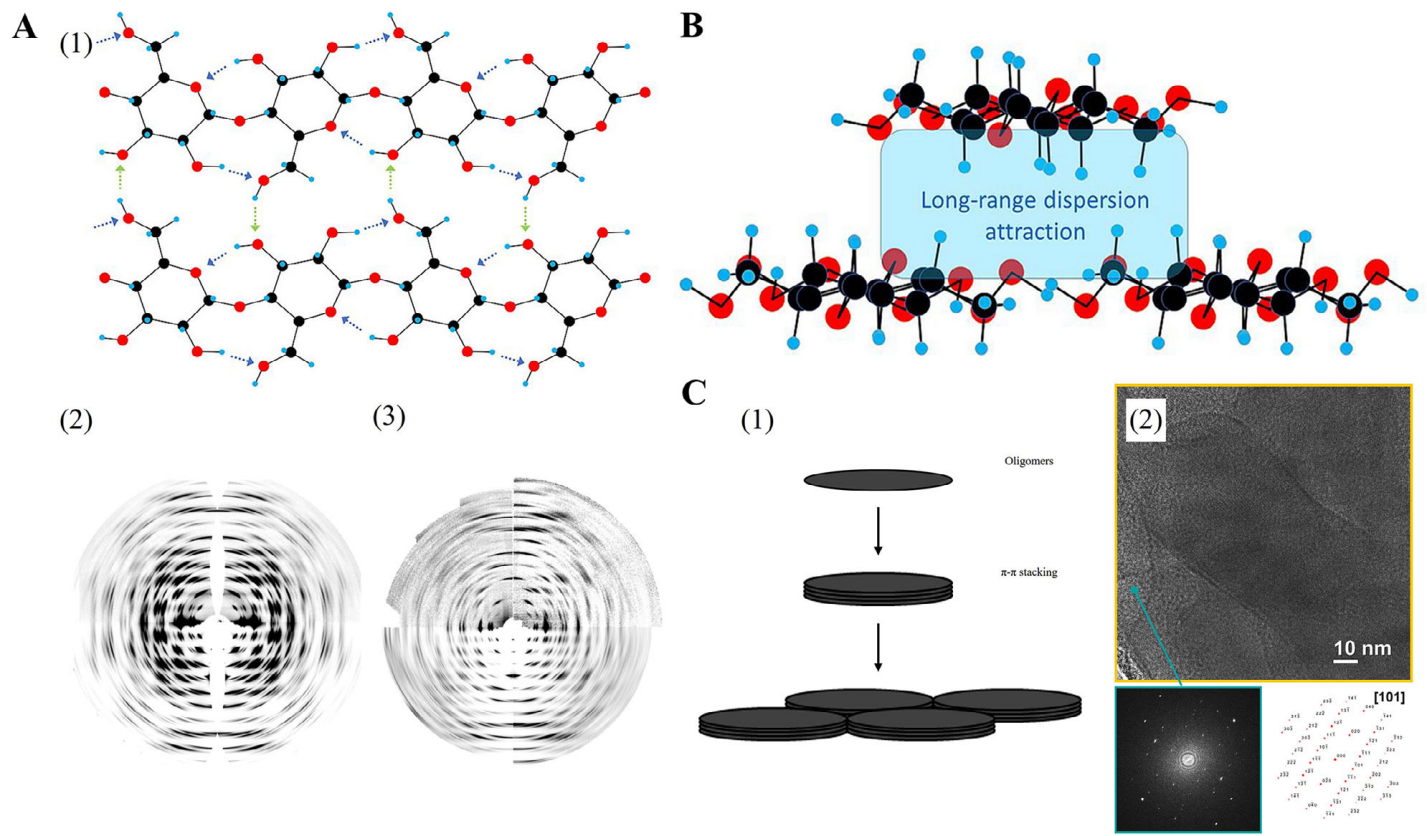
Overview of molecular structure formation. (A) (1) H‐bonding in cellulose Iβ, showing intramolecular (blue arrows) and intermolecular (green arrows) H‐bonds [222]. Reproduced under the terms of the CC BY 4.0 license from ref [222], Copyright 2023, The Authors. (2, 3) Synchrotron XRD data (2) and neutron fiber diffraction patterns (3) of *Halocynthia* cellulose Iβ [54]. Reproduced with permission from ref [54], Copyright 2002, American Chemical Society. (B) VdW force between cellulose Iβ sheets [222]. Adapted under the terms of the CC BY 4.0 license from ref [222], Copyright 2023, The Authors. (C) (1) Schematic of oligomers stacked using π–π interactions. (2) TEM image of a thin, electron‐transparent region within an otherwise irregular eumelanin layer. Insets: FFT spot pattern and the corresponding electron diffraction simulation along the [97] zone axis [230]. Adapted under the terms of the CC BY 4.0 license from ref [230], Copyright 2025, The Authors, published by the Royal Society of Chemistry. **Abbreviations**: TEM, transmission electron microscopy; SAED, selected‐area electron diffraction; H‐bonds, hydrogen bonds; VdW, Van der Waals forces; FFT, Fast Fourier Transform; CC, Creative Commons.

As introduced in Section 2.2, chitin's acetamido groups enhance intra‐ and intermolecular H‐bonding between neighboring polymer chains [101, 102]. α‐, β‐, and γ‐chitin differ in their molecular arrangement within the lattice, influencing the crystal system and unit‐cell parameters. In these structures, chitin molecules form fibrils through H‐bonds, which are essential for the sheet‐like structure. α‐chitin, the most common form, exhibits strong intra‐ and intermolecular H‐bonding owing to its antiparallel arrangement [231]. The α‐chitin crystal lattice adopts an orthorhombic P2_1_2_1_2_1_ unit cell [232]. In β‐chitin, parallel polymer chains form one H‐bond per unit cell within a monoclinic P2_1_ lattice [233]. γ‐chitin, rare in nature, exhibits mixed chain orientation and forms a monoclinic P2_1_ unit cell [234]. In α‐ and β‐chitin, each chain forms hydrogen‐bonded sheets connected by strong interchain C‐O···HN H‐bonds and has O‐3’···H‐O‐5’ intrachain H‐bonds [235]. Unlike β‐chitin, which lacks intersheet H‐bonds, α‐chitin has weak interchain H‐bonds involving the C6’‐OH groups [231, 233]. Consequently, compared to α‐chitin, β‐chitin has weaker intermolecular forces and higher chemical reactivity [236].

Beyond polysaccharides, H‐bonds are key determinants of protein properties. While α‐ and CBPs share similar amino acid sequences, they differ markedly in their secondary protein structures. In keratins, H‐bonds stabilize the α‐helix by linking carbonyl oxygen to the hydrogen of an amino acid four residues away. This interaction twists the chain into a helix, with each turn of the α‐helix containing 3.6 amino acid residues [237]. The dimer, or coiled coil, consists of two α‐helices linked by intramolecular disulfide bonds [238, 239]. In contrast, feather CBPs are composed of β‐pleated sheets, formed by laterally packed β‐strands arranged parallel or antiparallel (the latter being more stable). The β‐pleated sheet is stabilized by H‐bonds between backbone atoms of neighboring β‐strands, either within the same polypeptide or between distinct chains, creating the sheet structure. Additionally, peptide bonds contribute to the formation of pleated configurations [148, 240]. Overall, variations in hydrogen‐bonding networks influence molecular packing, structural stability, and the higher‐order assembly of cellulose, chitin, and protein‐based biomaterials.

#### 3.1.2 Van der Waals Force

VdW forces, along with H‐bond, critically stabilize structures through intra‐ and interchain interactions. VdW forces describe atomic or molecular attractions and repulsions and are classified into three types: Keesom, Debye, and London dispersion forces. Keesom forces arise from differences in permanent charge distribution between molecules. Debye forces occur when a polar molecule induces a charge distribution in an adjacent non‐polar molecule. London dispersion forces result from induced dipoles created by instantaneous fluctuations in molecular charge distribution. These forces are important because they represent attractive vdW contributions among nonpolar molecules, and all materials are polarizable.

While H‐bonds have traditionally explained cellulose interactions, other forces, including vdW forces, are increasingly recognized [241]. VdWs forces contribute to stacking interactions that stabilize cellulose layers (intersheet interactions) (Figure 6B) [223] and also support intrasheet stability [242]. Using quantum chemical calculations, Parthasarathi et al. show that cellulose Iβ stability is derived from strong intrasheet H‐bonds and highly cooperative intersheet stacking interactions [243]. Energy calculations on the minimized cellulose Iβ structure show that its crystalline stability reflects a balance between electrostatic and vdW interchain interactions, with H‐bond accounting for ∼70% of the interchain electrostatic energy [244]. In α‐chitin, molecular simulations similarly show that crystal cohesion reflects vdW and electrostatic contributions, while chain binding is strengthened by H‐bonding and dispersion interactions [245].

Quantifying these forces is essential for understanding their effect. Nishiyama estimated the interchain H‐bond energy in cellulose Iβ to be ∼24 kJ/mol based on evaporation enthalpy trends of alkyl alcohols and alkanes [246]. In contrast, the London dispersion interaction was calculated at ∼67 kJ/mol per glucose molecule, indicating a dominant contribution (50–70%) to cellulose cohesion. Li et al. estimated intra‐ and interchain London dispersion interactions in cellulose and chitin to range from 45–74 kJ/mol per pyranose ring. These values exceed upper‐limit estimates for inter‐ and intrachain H‐bonds (27–50 and 21–53 kJ/mol, respectively) [247]. Thus, vdW interactions act cooperatively with H‐bonds to stabilize biomolecular assemblies and can make substantial contributions to the cohesion of polysaccharide structures.

#### 3.1.3 π–π Stacking

π–π stacking interactions, between aromatic rings with π orbitals, are a type of dispersion force arising from vdW forces [248, 249]. These interactions cause stacking in small and large molecules, with typical distances of 3–4 Å, playing a key role in chemistry and biology [250, 251, 252, 253].

The chemical structure of eumelanin is difficult to quantify due to its insolubility; however, its hierarchical structure has been studied more extensively than that of pheomelanin [254, 255, 256, 257, 258]. The stacked oligomer model of eumelanin is widely accepted [259, 260], proposing that eumelanin particles consist of protomolecular structures, each containing four to five planar sheets. These sheets contain four to eight 5,6‐dihydroxyindole units, primarily stabilized by π–π stacking (Figure 6C[1]) [261, 262]. TEM combined with electron diffraction analysis identified a preferential crystallographic orientation of eumelanin along the [97] zone axis, indicating localized crystalline order within the nanostructure (Figure 6C[2]) [230]. These π–π interactions provide a key molecular driving force for the stacking and higher‐order organization of eumelanin oligomers into particulate structures.

### 3.2 Macroscopic Formation for Photonic Nanostructures

#### 3.2.1 Phase Separation

Phase separation, a thermodynamic process, occurs when a molecular species reaches saturation, demixing into dilute and dense phases, which are crucial in various physical phenomena. In biological systems and polymer science, liquid–liquid phase separation (LLPS) drives the segregation of a single liquid into polymer‐rich and polymer‐depleted phases [263, 264, 265, 266, 267].

Phase separation occurs through two primary pathways: nucleation and growth (NG) and spinodal decomposition (SD) (Figure 7A[1]). NG are activated processes occurring when a system moves from a stable to a metastable region in the phase diagram, with diffusion along the concentration gradient from high to low. In contrast, spinodal decomposition is a spontaneous process where a homogeneous phase becomes thermodynamically unstable, uniformly decomposing into two equilibrium concentration phases. The theoretical foundation of spinodal decomposition was developed by Cahn, Hilliard, and Cook [268, 269]. The detailed mathematical properties of the model are reviewed by Fife [270]. The similarity between photonic nanostructures in organisms and the morphologies seen in LLPS suggests that these nanostructures form through phase separation. Bird feather barbs, containing CBPs and air nanostructures, are proposed to self‐assemble in the cytoplasm of medullary cells through phase separation and kinetic arrest [135, 159]. These structures exhibit diverse morphologies, including channel, sphere, and fiber nanostructures [271]. Channel nanostructures, resembling those in SD, consist of CBPs and tortuous air channels. Sphere nanostructures, resembling those in NG, comprise quasi‐periodic, closely packed spherical air cavities, each surrounded by a CBP matrix (Figure 7A[2]) [125]. Prum et al. investigated the development of channel nanostructures in blue and yellow macaws (*Ara Ararauna*) using TEM, observing quasi‐ordered photonic structures forming after cell death, when cytoplasmic voids, nuclei, and other cellular machinery were filled with air [159]. Additionally, a single gyroid structure in the barb of the blue‐winged leafbird is proposed to form through the same process [272]. Burg et al. studied Cyphochilus and Lepidiota stigma, known for producing the brightest structural whites in nature and characterized by a disordered α‐chitin network (Figure 7A[3]) [118]. Using X‐ray nanotomography and finite‐difference time‐domain (FDTD) methods, they replicated the morphology and optical properties of beetle scales formed through SD, employing optical simulation and synthetic mimics. These studies support LLPS in forming intricate natural nanostructures.

**FIGURE 7:**
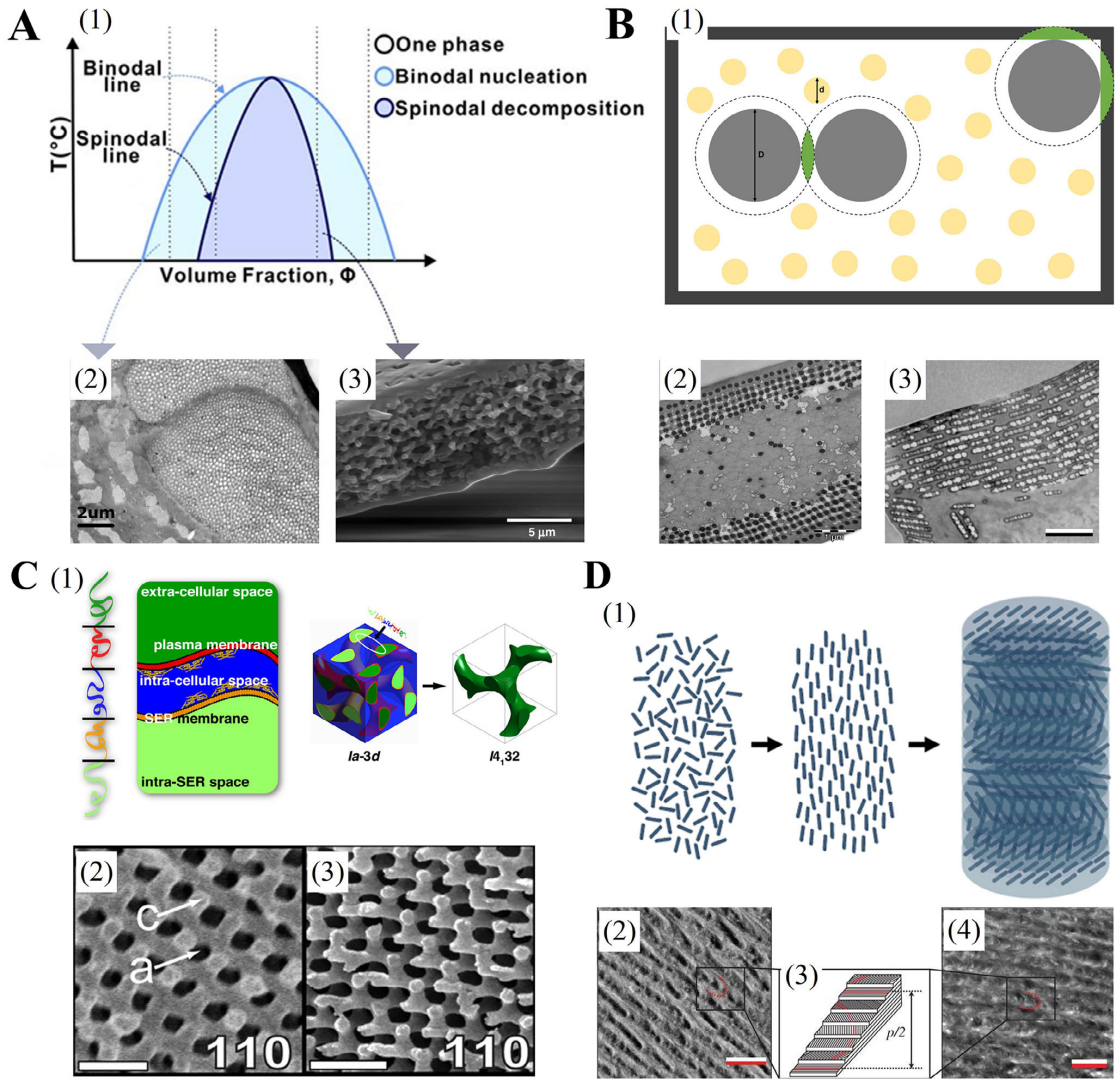
Overview of macroscopic formation mechanisms for photonic nanostructures. (A) (1) Schematic of phase separation in a simple two‐component system featuring a phase diagram [273]. Adapted under the terms of the CC BY 4.0 license from ref [273], Copyright 2021, The Authors. (2) Sphere‐type spongy CBPs nanostructure in the barb of Cotinga maynana imaged using TEM [125]. Adapted with permission from ref [125], 2011 Optical Society of America. (3) Disordered chitin network in the white beetle (*L. stigma*) [118]. Adapted under the terms of the CC BY 4.0 license from ref [118], Copyright 2019, The Authors. (B) (1) Schematic of overlapping excluded volumes of large particles at different positions within the container. (2) Ordered melanosome array in an adult peacock (*Pavo cristatus*) eyespot barbule imaged using TEM [274]. Adapted with permission from ref [274], 2011 Optical Society of America. (3) Multilayered melanosome structure in an Anna's hummingbird (*Calypte anna*) barbule imaged using TEM [275]. Adapted under the terms of the CC BY 4.0 license from ref [275], Copyright 2018, The Authors. (C) (1) Schematic of self‐assembly in arthropod photonic nanostructures and phase separation in block copolymers. Reproduced with minor modifications [212]. Reproduced with permission from ref [212], Copyright 2015, American Chemical Society. (2, 3) Structural color and photonic nanostructures of arthropod (2) *Platyaspistes venustus* (Curculionidae), single gyroid (I4132); (3) *Eupholus quintaenia* (Curculionidae), single diamond (Fd3̅m) and face‐centered orthorhombic (Fddd). Scale bars: 25 µm (1); 50 µm (2); and 500 nm (3, 4). (EN, chitin; c, air void; a). Reproduced with minor modification [212]. Adapted with permission from ref [212], Copyright 2015, American Chemical Society. (D) (1) Schematic of liquid crystal self‐assembly [276]. Adapted under the terms of the CC BY 3.0 license from ref [276], Copyright 2022, The Authors. (2,4) Helicoidal structure in *Margaritaria nobilis* fruit, with (3) schematic illustration [35]. (EN, pitch; p) Scale bars, 200 nm (2) and 500 nm (4). Adapted under the terms of the CC BY 4.0 license from ref [35], Copyright 2016, The Authors. **Abbreviations**: TEM, transmission electron microscopy; SAED, selected‐area electron diffraction; CC, Creative Commons.

#### 3.2.2 Depletion Attraction

The depletion effect was first described by Asakura and Oosawa in the 1950s [277, 278]. The stability of colloidal systems containing large, hard particles with a diameter D suspended in a solution is influenced by small, non‐adsorbing particles or polymers of diameter d, known as depletants (Figure 7B[1]). Since depletants cannot penetrate the large particles, an excluded volume of radius d/2 forms around each large particle [279]. When the excluded volume around large colloidal particles does not overlap, depletion‐induced osmotic pressure is isotropic, and electrical repulsion prevents particle aggregation [280]. However, as the large particles approach each other, their excluded volumes overlap, increasing depletant entropy by expanding accessible volume. This creates an osmotic pressure imbalance that drives the large particles together, producing the depletion effect [281].

Early research on iridescent Selaginella leaves with opal structure suggests that colloidal crystal formation is driven by depletion attraction [282]. During this process, a long‐chain polymer serves as a depletant, aiding the formation of photonic structures through osmosis. In bird feather barbules, iridescent coloration arises from the ordered arrangement of melanosomes into arrays or multilayered structures (Figure 7B[2,3]) [274, 275]. These melanosomes move from melanocytes into barbule cell keratinocytes [283]. According to Maia et al., iridescent feather nanostructures self‐assemble through depletion attraction, with forces pinning melanosomes to one another and to the barbule cell wall [203]. Comparative studies of iridescent and non‐iridescent barbule development in the blue‐black grassquit (*Volatinia jacarina*) support this hypothesis and are consistent with reports of iridescent feathers in wild turkeys (*Meleagris gallopavo*) and hummingbirds (*Calypte anna*) [204, 284]. Depletion attraction therefore provides an entropy‐driven mechanism by which colloidal components such as melanosomes can become spatially organized into photonic structures.

#### 3.2.3 Lipid Bilayer Self‐Assembly

Crystal‐like structures in some butterflies and arthropods are thought to arise from self‐assembly within a cellularly established lipid bilayer template. Michielsen and Stavenga [285] show that the cuticle structures of some papilionid and lycaenid butterflies can be modeled as a gyroid, suggesting that butterfly photonic crystals, composed of chitin and air nanostructures, form through self‐organizing physical dynamics within biological lipid‐bilayer membranes, producing single gyroid (I4_1_32) nanostructures [81]. Initially, a core–shell double gyroid $\left(\mathrm{Ia}\bar{3}d\right)$ template is formed, which, through chitin deposition and cell drying, transforms into a single gyroid nanostructure. Saranathan et al.  [212] analyzed various photonic nanostructures and provided developmental insights by comparing them to the microphase separation in block copolymers (Figure 7C). This includes butterfly wing scales from two families [81] with single gyroid (I4_1_32) photonic crystals (Figure 7C[2]), and cover scales of a fossil weevil, exhibiting single diamond $\left(\mathrm{Fd}\bar{3}m\right)$ photonic crystals (Figure 7C[3]), determined using synchrotron small‐angle X‐ray scattering. Their findings indicate that arthropod photonic nanostructures, formed through self‐assembly of infolding lipid‐bilayer membranes, closely resemble the morphologies of amphiphilic macromolecules. These studies suggest that cellular membrane organization can act as a physical template that guides chitin deposition into complex three‐dimensional photonic architectures.

#### 3.2.4 Liquid Crystalline Self‐Assembly

Cellulose and chitin nanofibrils form chiral nematic (also termed cholesteric) liquid crystals with helicoidal organization [286], and in cellulose‐based plant systems, such structures generate structural color in some fruits through helicoidal arrangement of cellulose in the cell wall (Figure 7D[2–4]) [19, 35, 287]. Based on structural similarities, the liquid‐crystalline self‐assembly model has been widely proposed as a plausible mechanism (Figure 7D[1]) [211], and is supported by efforts to replicate similar structures using cellulose nanocrystals (CNCs) and chitin nanocrystals extracted from natural materials [79, 276, 288]. Structurally colored films with chiral nematic morphology are typically produced by evaporating CNC suspensions [289]. Related cellulose‐derived systems, such as hydroxypropyl cellulose, likewise exhibit concentration‐dependent cholesteric liquid‐crystalline self‐assembly [290]. Helicoidal structure formation depends on a balance of attractive and repulsive forces [213]. Final nanostructures are shaped by water loss between and within CNCs, which is influenced by humidity [291]. Additionally, temperature, pressure, and other factors can influence the optical and mechanical properties of the final structures [292]. Thus, liquid‐crystalline self‐organization provides a physicochemical route to helicoidal photonic architectures, although direct confirmation of the corresponding assembly process in vivo remains limited. Recent studies focus on quantifying forces and elucidating atomic‐level interactions. Many studies have applied structural similarities to explore developmental processes, but confirming these processes through such comparisons remains challenging. Advancements in research methods enabling real‐time, in vivo observation can substantially bridge gaps in our understanding.

## 4 Computational Approaches

The hierarchical processes described in Sections 2 and 3 span multiple length and time scales, requiring computational tools spanning multiple scales. Furthermore, understanding molecular structure is challenging because biological polymers are long, and their formation is influenced by environmental factors and substances within the cell. This complicates describing in vivo nanostructure formation, as it arises from interactions among complex biomolecules. Therefore, computational methods, including physical simulations and artificial intelligence (AI), have been proposed to elucidate biological nanostructure formation across multiple scales. This section reviews computational studies elucidating nanostructure formation in biomaterials with structural color.

### 4.1 Physical Simulations

Physical simulations are classified based on the scale of the modeled elements. This is because the dominant forces vary with element size, leading to different governing equations [293]. Therefore, physical simulations of nanostructure formation are discussed in terms of atomic, molecular, and continuum scales.

#### 4.1.1 Atomic Simulation

Biomaterial structures can be predicted by calculating the electronic structures of atomic models. First‐principles quantum mechanics is required to calculate the electronic energy states of the system. Calculating the energy of a many‐body system requires numerous computational resources as the number of electrons increases. Density functional theory (DFT) enables efficient calculations of the system energies based on the Hohenberg–Kohn (HK) theorem, through the use of electron pseudopotentials and exchange–correlation functionals. A pseudopotential approximates the inner‐core electron density using uniform, plane wave, Gaussian, projector‐augmented wave, and localized atomic orbital functions [294, 295, 296, 297, 298, 299]. This effectively reduces computational cost because core electrons have complex wave functions, whereas the atomic interactions are governed primarily by valence electron wave functions. The reliability of several pseudopotentials was verified by comparing DFT results with all‐electron calculations [297, 299, 300, 301, 302]. The Kohn–Sham equation, formulated within the HK framework, enables calculations of electron density and total energy using exchange–correlation functionals. The exchange–correlation concept includes electron repulsion caused by Pauli exclusion (exchange) and Coulomb force (correlation), but its exact formula is generally unknown for electronic structures. Therefore, various approximations, including local density and generalized gradient approximations, have been developed to model the exchange–correlation functionals [294, 295, 296, 303, 304, 305].

Nishiyama et al.  [306] suggest two fundamental H‐bonding networks observed in cellulose Iβ using the Perdew–Burke‐Ernzerhof (PBE) functional, which is widely used for approximating the exchange–correlation functional in solid‐state DFT calculations. Based on the findings of Nishiyama et al., Lee et al.  [307] derived energy‐minimized networks of cellulose Iα and Iβ crystals using a DFT approach with dispersion corrections (Figure 8A). Hayakawa et al.  [308] investigated possible cellulose Iβ and II crystal models based on the H‐bonds proposed by Nishiyama using DFT calculations with the PBE and dispersion correction functionals. As DFT calculations provide the electronic structures of the input system, spectroscopic properties can be estimated through subsequent computations. Therefore, comparing the spectroscopic data with computationally estimated spectra can further validate the reliability of DFT‐calculated molecular structure. Barsberg et al.  [309] developed a method to predict Fourier transform infrared spectra (FT‐IR) based on DFT calculations of cellulose Iα and Iβ chains. Kubicki et al.  [310] and Yang et al.  [311] used DFT calculations for three 18‐chains cellulose microfibrils found in plant cells to derive nuclear magnetic resonance (NMR) chemical shifts and wide‐angle X‐ray scattering diffractograms. In both studies, the DFT‐calculated spectra were validated against experimental measurements. Given the reliability of DFT for spectral calculations, Wang et al.  [312] identified multiple interior and surface cellulose structures in the primary cell walls of dicot and monocot plants and combined NMR analysis with DFT to elucidate detailed cellulose polymorphism within plant primary cell walls. In a subsequent study, Yadav, Asha et al. [313] further clarified the hydrogen arrangements and associated optoelectronic properties of cellulose Iα and Iβ.

**FIGURE 8:**
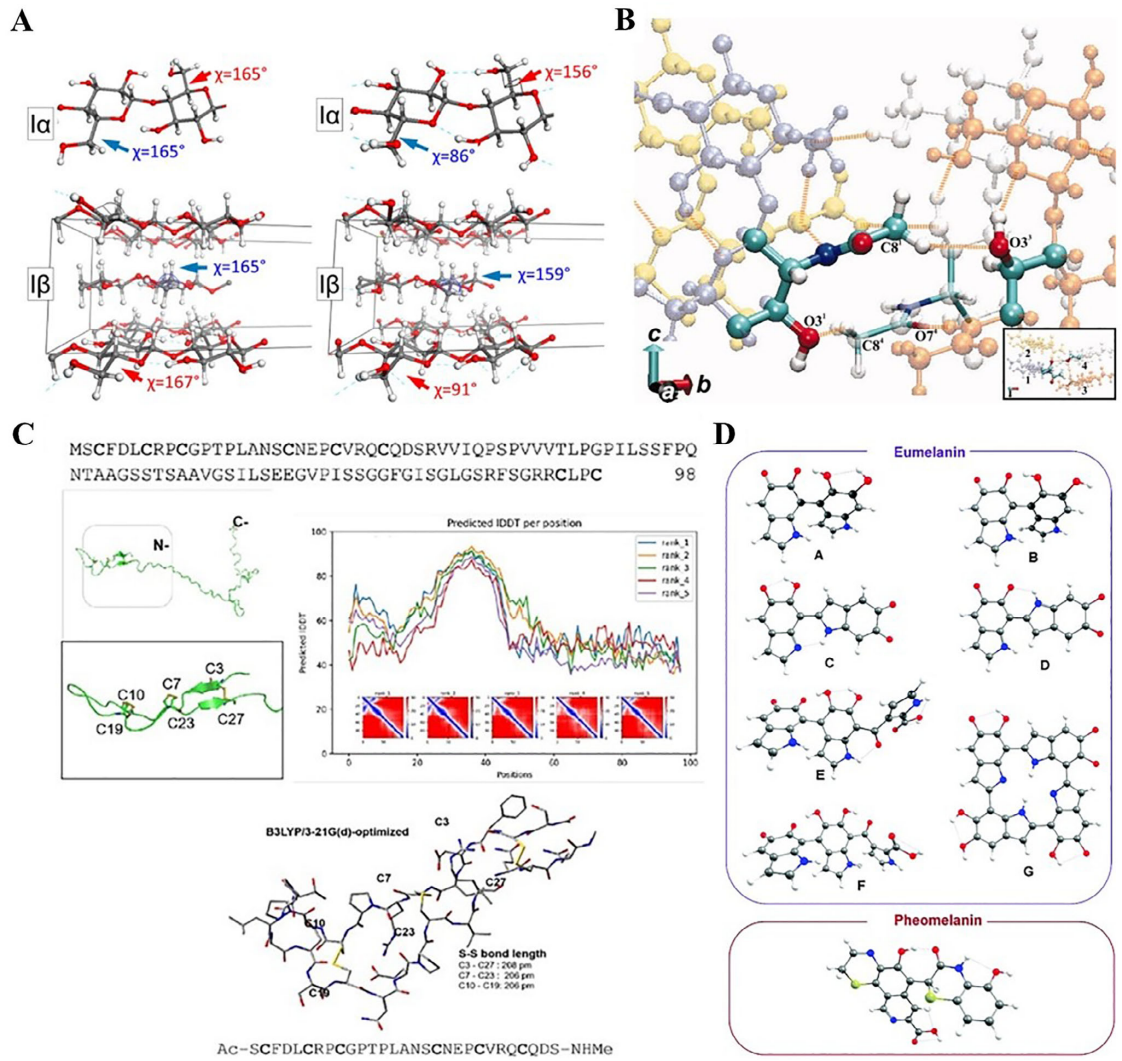
DFT simulations of stabilized biomolecular structures. (A) Energy‐minimized structures of cellulose crystals Iα and Iβ, calculated by DFT from two basic configurations. Reprinted with permission [307]. Reproduced with permission from ref [307], Copyright 2013, American Chemical Society. (B) Stabilized morphology of the acetylamino chain network in the ground state α‐chitin crystal. The H‐bonds indicated as C8‐H…O3 contribute to the energy minimization of the acetylamino group [314]. Reproduced with permission from ref [314], Copyright 2013, John Wiley & Sons. (C) The N‐terminal molecular structure of CFK was predicted using AlphaFold2 software and optimized with DFT calculations [315]. Reproduced with permission from ref [315], Copyright 2023, Elsevier. (D) Optimized structures of eumelanin and pheomelanin oligomer candidates. Ground state conformers with minimum energy were selected through DFT calculations, and their optical properties were compared [316]. Reproduced under the terms of the CC BY 3.0 license from ref [316], Copyright 2016, The Authors, published by the Royal Society of Chemistry. **Abbreviations**: DFT, density functional theory; CC, Creative Commons; CFK, Chicken Feather Keratin.

For α‐chitin, Petrov et al.  [314] predict potential crystal geometries using DFT with Troullier–Martins pseudopotentials (Figure 8B). They propose thermodynamically and energetically stable models and validate the calculated IR spectra against experimental results. Building on these findings, Deringer et al.  [317] report two distinct crystallographic motifs of α‐chitin based on hydroxymethyl group orientation. These motifs are essential for constructing a stable α‐chitin tetramer and are subsequently used to estimate its mechanical properties [318]. Li et al.  [247] calculated the strength of the London interaction and H‐bond in α‐chitin using DFT analysis with the motifs.

Tuly et al.  [315] conducted DFT calculations to estimate the molecular structure and Raman spectra of Chicken Feather Keratin (CFK) 1 (Figure 8C). While CFK exhibits a complex structure comprising 98 amino acids, only 28 are included in the calculations. A supercomputer system capable of handling high computational demands is required because the 28 amino acids constitute long polypeptides, making DFT calculations challenging using conventional processors. By correlating with DFT studies of dipeptides [319, 320, 321] or tripeptides [322, 323, 324, 325], further insights can be obtained by analyzing longer polypeptides without computing larger atomic arrays. However, the entire electronic structure of the system changes continuously as atoms are added.

The optimized structures of eumelanin monomer [326, 327, 328, 329, 330, 331, 332], dimers [199, 330, 331, 332, 333], trimers [316, 333], tetramers [199, 316, 333, 334, 335, 336], pentamers [334, 335], and hexamers [335], along with their formation mechanisms, are investigated through DFT calculations. Further studies are needed for larger molecules because the force between extended eumelanin oligomers drives the formation of eumelanin protoparticles through oligomer stacking in melanosomes. In contrast, pheomelanin is not a long‐chain polymer and primarily exists as a group of diverse molecules. Marchetti et al.  [316] and Kishida et al.  [337] report optimized molecular structures and formation mechanisms of pheomelanin components using DFT analysis (Figure 8D). Based on the electronic structures obtained from DFT calculations, broadband light absorption mechanisms of melanin molecules are also elucidated [326, 329, 333, 338]. Cardia et al.  [339] offer insights into the absorbing subunits of eumelanin by investigating the absorption spectra of eumelanin tetrameric molecules using time‐dependent DFT.

Other atomic simulation methods, including the quantum theory of atoms in molecules and the Hartree‐Fock method, are employed to calculate the electron density of cellulose molecules and oxidized eumelanin, respectively [340, 341]. However, DFT provides high accuracy at significantly lower computational costs, making these methods less common and largely limited to specific applications. In addition, DFT simulators are further developed owing to their widespread use, encompassing specific applications typically addressed by other simulation methods.

#### 4.1.2 Molecular Simulation

Atomic simulations are effective for calculating monomers or short chains of cellulose, chitin, CBPs, and melanin. However, applying these methods to larger molecules requires significantly higher computational cost. Therefore, molecular dynamics (MD) simulations, which apply Newton's laws of motion, are essential for molecular‐scale modeling. MD can be classified into classical MD and ab initio MD (AIMD); classical MD models atoms as classical particles, while AIMD accounts for the motions of electrons and nuclei. AIMD is often combined with atomic simulations, including DFT, enabling more accurate calculations than classical MD [342, 343, 344, 345]. For MD calculations, the design constraint and force field must be selected first, based on the specific challenge to be addressed. The constraint ensemble defines the controlled variables used to calculate the movement of atoms on a sufficient timescale within a given computational cost. For the microcanonical (NVE), canonical (NVT), and isothermal isobaric (NPT) ensembles, the abbreviations N, V, E, T, and P denote conserved moles, volume, energy, temperature, and pressure, respectively [344, 346, 347, 348, 349, 350]. For example, under the NVT ensemble, the moles, volume, and temperature of the system remain constant. The force‐field, also known as the interatomic potential, is developed from theoretical approximations and experimental results [351, 352]. Therefore, force fields exhibit substantial diversity based on the materials, bond types, functionalities, and applications. Starting from the classical force‐field, these models can be grouped into polarizable, coarse‐grained, or machine‐learning (ML) force fields [351, 353]. However, this classification is not rigid, as force‐field types are diverse and multiple force‐fields can be combined.

Heiner et al.  [354] conducted MD simulations of cellulose Iα and Iβ under NVT using the saccharide force‐field GROMOS87, providing the generalized structures and energetics of both allotropes and report that cellulose Iβ is thermodynamically favorable. Hardy et al.  [355] performed MD simulations under NVE by initializing cellulose allotropes using a diffraction‐based model. Through observing the behaviors of the initial structures, including torsional motion, isomerization, H‐bond breakage and formation, and chain movements, they propose an Iα to Iβ phase transition mechanism known as the break‐slip model. Several researchers, including Kroon–Batenburg, conducted MD calculations of cellulose crystals under NPT to characterize their mechanical properties [356, 357, 358, 359]. Mazeau et al.  [360] performed MD simulations under NPT to compare crystalline and amorphous cellulose, providing detailed structures with the conformational parameters and intermolecular interactions, reporting that solid modeling by constraining volume does not ensure mechanical stability. Kulasinski et al.  [361] report NPT‐based MD simulations of paracrystalline cellulose by comparing its crystalline and amorphous structures. Haigler et al.  [362] used MD with six glucans to investigate the mechanism of cellulose microfibril formation by the CSC. Saitoh et al.  [363] modeled larger cellulose molecules, cellulose nanofibers (CNF), and CNF microfibrils using coarse‐graining with a united‐atom method. Wan, Jia, and Chuanfu Luo employed steered MD simulations to investigate the response of parallel cellulose chains to force applied at different speeds [364]. Various coarse‐grained models of cellulose have been developed for MD calculations, based on the molecular scale, solvent environment, and applications (Figure 9A) [365, 366, 367, 368, 369, 370, 371, 372]. Nikolov et al.  [373] performed AIMD using an empirical force‐field under NPT to estimate the structure and elastic modulus of α‐chitin. The α‐chitin conformation space is calculated based on energy minimization and H‐bond maximization criteria. Petrov et al.  [314] report a larger α‐chitin crystalline model using the same methodology, exploring 108 structures to determine the ground‐state conformation of the H‐bond network. Jin et al.  [374] employed steered MD to investigate the molecular interactions between chitin and proteins, as proteins directly control chitin structure formation in vivo. Beckham et al.  [375] modeled the equilibrium crystal structure using 1,120 GlcNAc units, performing MD simulations sequentially under NVT and NPT (Figure 9B). Střelcová et al.  [376] provided eleven α‐chitin nanofibril structures using the GLYCAM06g force‐field to elucidate nanofibril self‐assembly movements. Salavati [377] employed reactive force‐field MD simulations to examine the properties of α‐ and β‐chitin and chitosan in aqueous environments, demonstrating that the high mechanical stiffness of α‐chitin arises from its highly crystalline molecular structure. At the molecular level, reactive force field MD simulations are also used to elucidate the structure‐property relationships of α‐ and β‐chitin crystals under tensile and shear deformations [378].

**FIGURE 9:**
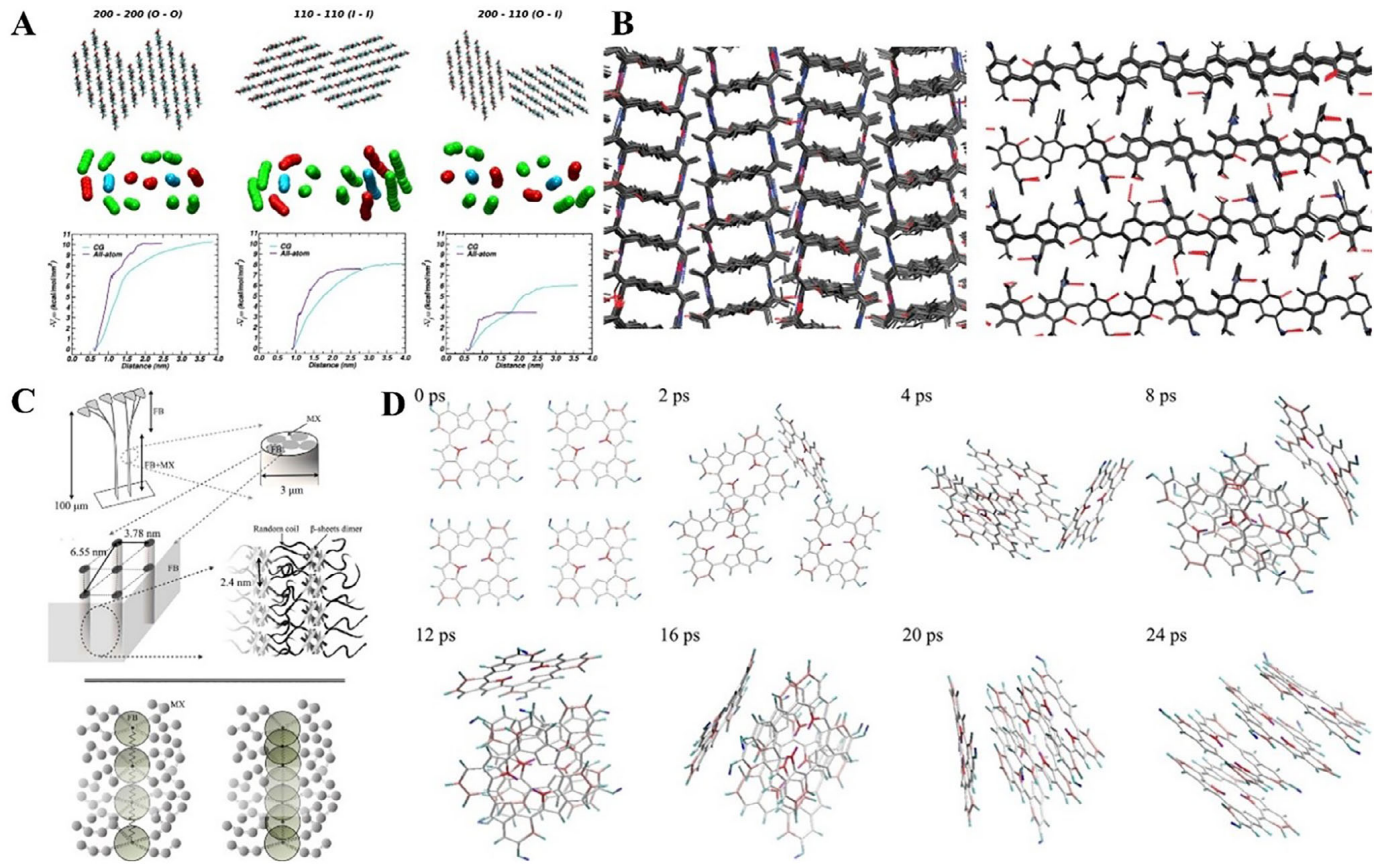
MD simulations enable efficient calculation of large‐scale molecules with high computational efficiency. (A) A coarse‐grained model of cellulose developed for efficient and accurate representation of the mean force potential [365]. Reproduced under the terms of the CC BY 4.0 license from ref [365], Copyright 2020, The Authors. (B) H‐bond networks schematics along the a‐ and c‐axes obtained from large‐scale MD simulations of α‐chitin. Reproduced with permission from ref [375], Copyright 2011, American Chemical Society. (C) A coarse‐grained model developed based on the molecular structure of gecko feet CBPs [379]. Reproduced with permission from ref [379], Copyright 2018, American Chemical Society. (D) Self‐assembly of eumelanin tetramers from optimized structures analyzed through MD simulations [380]. Reproduced with permission from ref [380], Copyright 2013, American Chemical Society. **Abbreviations**: CC, Creative Commons; MD, molecular dynamics; CBPs, corneous beta‐proteins; H‐bond, hydrogen bonds.

Calvaresi et al.  [122] report the structures of CBPs and the filament polymerization process in five sauropsids through molecular simulations. Using Quark software, the 3D structures of the proteins are first predicted, and the most favorable monomeric, dimeric proteins, and β‐fibril formation mechanisms are investigated using a docking protocol. Subsequently, MD simulations offer more stable protein structures. Müller‐Plathe et al. employed MD to analyze the CBP structure of the gecko among the sauropsids [379, 381, 382, 383, 384]. The coarse‐grained and united‐atom model of gecko CBPs is developed for MD simulation to examine larger keratin molecules, and the surface adhesion mechanism of geckos is investigated from molecular to mesoscale (Figure 9C) [379, 381, 382]. Using the developed coarse‐grained model, Materzok et al. conducted force probe MD by constructing four layers of CBP nanofibrils [383]. Eslami et al.  [384] modeled the interactions between 18 chains of amorphous CBPs and water molecules under varying relative humidity. The united‐atom force‐field GROMOS54A7 and the grand canonical ensemble are employed for MD simulation, as this ensemble allows water molecule exchange in the hydrated keratin system.

Based on first‐principles DFT calculations of eumelanin monomers, Antidormi et al.  [385] report an MD simulation that illustrates the time‐dependent polymerization of eumelanin monomers. The average chain lengths of the eumelanin oligomers converged, indicating that the number of molecules decreases exponentially with increasing chain length. Chen et al.  [327, 341] suggest possible molecular structures of eumelanin from dimers to tetramers based on AIMD, demonstrating that 1) planar and cyclic structures ensure stability, and 2) oligomers with larger molecular weights are less favorable. Additionally, Chen [380, 386] modeled the self‐assembly of eumelanin protomolecules using all‐atom MD with a consistent valence force‐field (Figure 9D). Large‐scale modeling allows for comparison of the resulting mechanical and optical properties with experimental results. Soltani et al.  [387, 388] investigated the physical mechanism underlying eumelanin tetramer stacking by varying its concentration in water over extended timescales. In addition, the aggregated structure and H‐bond networks in the system are analyzed. Marchetti et al.  [316] investigated the structure of pheomelanin by comparing eumelanin and pheomelanin from monomer to tetramer structures using AIMD simulations. Their results indicate that the interactions between eumelanin and pheomelanin molecules require further investigation to elucidate the pheomelanosome formation mechanism. Valiyaparambil et al. analyzed the electronic structure and intrinsic reactivity of melanin molecules using DFT and investigated the dynamic interactions between melanin and proteins in biologically relevant environments through MD simulations [389].

#### 4.1.3 Continuum Simulation

Continuum simulations are used to model bulk‐scale materials, utilizing macroscopic properties within a mesh framework rather than considering interactions between molecular structures. Therefore, selecting appropriate parameter values and optimizing the spatial mesh design are critical for achieving accurate and convergent results. Continuum model algorithms can be classified based on their governing equations, solver approximations, and time‐dependencies. The governing equations are fundamental differential equations that should be solved within the framework of classical physics. For example, the Navier–Stokes and Maxwell equations are essential for modeling fluid dynamics and electromagnetic simulations, respectively. Representative continuum model solvers include the finite element method (FEM) and the finite difference method (FDM). In FEM, the modeling structure is first divided into polygonal mesh structures, and the geometrical space is approximated according to the mesh shape to solve the differential equations. In FDM, the differential equations are approximated using a linear combination of derivative terms from a Taylor series, making a cuboid mesh structure the most favorable for implementation. For simulating a time‐dependent phenomenon, time‐domain FEM and FDTD solvers can be applied.

Most biochemical interactions can be studied using fluid simulations. However, the structure formation process of these materials has not been addressed in continuum‐level simulations, as the structure of the biomolecules and their biological environment remains incompletely understood. Directly simulating biological interactions that generate bulk‐scale photonic structures remains challenging. Therefore, several continuum modeling studies focus on analyzing bulk properties, providing insights for estimating the natural structure mechanisms underlying the formation of these structures. Mechanical characteristics are largely determined by physical interactions and bonding at the atomic and molecular scales [390, 391, 392, 393, 394, 395, 396, 397, 398, 399, 400]. Similarly, continuum modeling of the optical properties of photonic bionanostructures can be correlated, as these properties are affected by electronic states and nanostructures [177, 179, 401, 402, 403, 404, 405, 406, 407, 408, 409]. FEM and FDM can be applied to mechanical and optical simulations. For example, Siddique, Radwanul Hasan, et al. [410] employed 2D FEM to investigate the optical characteristics of nanostructures in blue Morpho butterflies, proposing that the wide angular blue reflection arises from the combination of multiple structural components. Similarly, Wilts, Bodo D., et al. [411] report that the 3D network morphology of the *Cyphochilus* beetle represents an evolutionary optimization for bright whiteness, using X‐ray tomography and FDTD simulations. Jeon, Deok‐Jin, et al. [131] conducted FDTD simulations with a 3D tomographic image to analyze how the thickness of spongy nanostructure affects structural color in Eurasian jay feathers. However, mechanical characteristics are primarily evaluated using FEM, while optical properties are commonly analyzed with FDTD, reflecting time dependency and software maturity. All physical simulations exhibit a trade‐off between computational cost and accuracy. Thus, appropriate methods and system optimization must be selected to obtain reliable results within available computational resources. A comprehensive understanding of nanostructure formation mechanisms across multiple scales is required. However, atomic stimulations such as DFT cannot be applied to very large systems, including proteins and macromolecules, owing to computational limitations. Therefore, correlating simulation results across the atomic, molecular, and continuum levels is crucial. Defining a representative unit cell for small‐scale simulation enables the extension of these results to larger scales, preserving the underlying physical principles. Nevertheless, there remains a need to model larger systems using small‐scale simulations to improve accuracy. To address the computational cost associated with large systems, quantum computing reduces the time complexity of fundamental algorithms. Time‐efficient DFT calculations have been demonstrated [412, 413, 414]. Quantum computing approaches have also been applied to MD calculations, especially for protein‐folding challenges [415, 416, 417, 418, 419, 420]. Quantum computers are advancing with more qubits and longer coherence times, enabling more gate operations with reduced noise.

### 4.2 Artificial Intelligence

Recently, AI technology has gained prominence with the development of artificial neural networks and learning techniques. Artificial neurons are designed to emulate the operating principles of the human nervous system. The field of study that employs artificial neurons is referred to as ML. Deep learning (DL), a subcategory of ML, has demonstrated outstanding performance across various applications through deep neural networks (DNNs) composed of multiple layers of artificial neurons. With the emergence of AI techniques, several studies have addressed technical challenges in understanding the mechanism of nanostructure formation. Since molecules in vivo are affected by numerous, sometimes unknown parameters, ML provides an efficient approach to predicting highly ordered nanostructures. In addition, ML‐based methods for searching, classification, and super‐resolution techniques can further aid in elucidating the formation mechanisms of photonic nanostructures.

AI techniques have been applied by some researchers to predict the formation of cellulose, chitin, keratin, and melanin nanostructures. Gao et al.  [421] developed an ML‐based algorithm that predicts holocellulose types using Raman features as input variables and measures holocellulose content as the output variable. Gupta et al.  [422] employed a support vector machine (SVM) to characterize the structure and function of cellulose synthase. The SVM is trained to extract unique features of the synthase from the polypeptide sequence, successfully classifying 306 proteins as cellulose synthase or non‐cellulose synthase. Lee et al.  [423] used a DNN to predict microbial activity of bacteria on polymeric chitin, or GlcNAc. The DNN is trained using spatiotemporal patterns generated from agent‐based Monte Carlo simulations. Banerjee et al.  [424] developed ML‐based classification models that extracted features from keratin amino acid sequences and categorized them as mammalian and non‐mammalian keratin. Choudhury et al.  [425] trained a DNN using data obtained from time‐domain DFT and MD simulations of melanin tetramers. Using the kernel‐ridge regression algorithm, the model can inversely assign melanin structures based on the input spectrum. Subsequently, the approach was extended to predict the structural parameters that determine the conformers within the heterogeneous biopolymer melanin [426]. Bosch et al.  [338] report a DNN capable of replacing thermodynamic and optical characteristic simulations using eumelanin dimer structure information as input.

Other AI‐based techniques also aid in elucidating the formation mechanisms of photonic nanostructures in nature. Spectrometric analyses, including NMR, FT‐IR, and mass spectroscopy, are enhanced by AI for baseline correction of the spectrum, rapid spectrum matching with libraries, peak‐shift detection, and rapid molecular structure prediction [427, 428, 429, 430, 431, 432]. In addition, DNNs improve imaging quality by increasing the resolution of micrographs of biological samples [433, 434]. Super‐resolution techniques for optical and electron microscopy enable the prediction of complex structures with high precision [435, 436, 437, 438]. Researchers develop DNNs to model physical phenomena or molecular structures as alternatives to physical simulations [439, 440, 441]. As a technical tool that enhances analytical capabilities, AI also provides considerable potential to identify the biological and physical mechanisms underlying in vivo nanostructure formation. When the analysis phenomenon is defined, a well‐trained DNN can provide accurate estimations in a relatively short time [442, 443, 444]. As a technical tool that enhances analytical capabilities, AI also provides considerable potential to identify the biological and physical mechanisms underlying in vivo nanostructure formation.

In particular, ML algorithms for protein structure prediction [445, 446, 447, 448, 449, 450, 451], most notably AlphaFold2 [452], can be applied to CBPs, enzymes, and other proteins involved in nanostructure formation. However, CBPs remain a prime example of proteins whose structures are difficult to predict using AI‐based tools due to their repetitive and β‐sheet‐rich sequence characteristics, and the reasons for this can be summarized into three main points. First, there is the issue of training data. Predictors, including AlphaFold2, are trained on experimentally determined structures to infer folding from sequence [452], but, to the best of our knowledge, no atomic‐level experimental structure of a CBP has been reported. Current structural models rely on modeling based on fiber X‐ray diffraction [14, 152] or ab initio prediction [122], so that fibrous β‐proteins comparable to CBPs are severely underrepresented in the training data. Second, there is the issue of sequence composition. Feather CBPs contain only ∼32 conserved residues that form the filament, whereas the remaining residues constituting the matrix are compositionally biased, repetitive sequences whose number and type vary considerably among species [14, 152]. Since regions with very low confidence in AlphaFold2 predictions largely overlap with such intrinsically disordered regions [453], AlphaFold2 predictions for chicken feather CBPs also assign high confidence only to the short central β‐sheet region and predict the flanking regions with low confidence (Figure 8C) [315]. Third, there is the problem of the prediction paradigm. Sequence‐based predictors rely on co‐evolutionary signals under the premise of “one sequence, one structure” [454], but the functional unit of CBPs is not a monomer but a filament formed by β‐sheet stacking of multiple chains [122, 152]. Just as it has been suggested that residue covariation in functional amyloids encodes the aggregated rather than the monomeric structure [454], the conservation pattern of the CBP gene family (Section 2.3), which contains many very similar paralogs, may also reflect the filament‐forming interface; therefore, dedicated training on the aggregated state would be required to utilize this for prediction.

While these limitations are difficult to resolve with ML alone, MD and ML precisely complement each other's weaknesses, making a hybrid approach combining the two methods particularly promising for CBPs. ML predictors rapidly provide plausible initial structures from sequences but fail to capture physical interactions or dynamics. MD explicitly addresses hydrogen bonding and van der Waals interactions discussed in Section 3, along with solvents, ionic conditions, and temperature, allowing for the description of β‐sheet stacking and filament nucleation, but on its own it cannot reach the spatiotemporal scale at which CBP filaments are organized. Therefore, the first direction is a flow from ML to MD. Using structures predicted by ML as an initial model for physics‐based MD refinement improves both global and local structural accuracy compared to using only ML predictions [455]. A similar strategy of docking predicted monomers and then stabilizing them with MD to obtain CBP dimers and β‐fibrils has already been applied to sauropsid CBPs [122]. In this process, the predicted structures can be verified using filament diameters and β‐sheet arrangements confirmed via fiber X‐ray diffraction as experimental constraints [14, 152]. The second direction is the flow from MD to ML. ML force fields trained with all‐atom MD data provide accuracy similar to ab initio methods at a much lower computational cost [353], and based on the same principles, coarse‐grained models parameterized from MD trajectories have already been used to link molecular‐level adhesion mechanisms with the mesoscale in gecko CBPs [379, 381, 382]. Extending this approach to feather CBPs opens the way to simulating how filaments and matrices undergo phase separation (Section 3.2) and organize into photonic nanostructures while maintaining molecular‐level details.

Beyond protein structures, sequence‐to‐function models are now expanding the scope of AI prediction to include the gene‐regulatory level that precedes assembly. For example, AlphaGenome takes up to 1 Mb of DNA sequence as input and predicts gene expression, splicing, chromatin accessibility, and genome contact maps, and scores the effects of variants by comparing the predicted values of the original sequence with the variant sequence [456]. Currently, it is trained only on human and mouse genomes and cannot be directly applied to birds, insects, or plants. However, if this model is expanded, it could become possible to link regulatory variants in melanogenesis‐related genes such as those encoding tyrosinase, TYRP‐1, and agouti signaling protein (Section 2.4), or the CBP gene cluster (Section 2.3), with expression programs that influence nanostructure formation. This would serve as the starting point for connecting the biological regulation and synthesis level presented in Section 2.5 and the structure‐level predictions in this section into a single prediction system.

Collecting thousands of datasets to train DNNs is time‐consuming and requires considerable effort, particularly in natural science. Therefore, few‐shot learning (FSL) and quantum machine learning (QML) algorithms, which can achieve high performance with fewer datasets, should be considered for future ML applications [457]. FSL is an ML task or algorithm that uses a limited supervised dataset for training [458]. FSL algorithms can address dataset‐collection challenges in training DNNs for biomolecular research through transfer learning, meta‐learning, or generative modeling. QML is implemented by processing quantum data through quantum gates within a quantum‐computing system [459, 460, 461, 462]. QML is expected to achieve quantum supremacy by reducing the number of datasets and iterations required to train the DNNs. Therefore, the integration of ML algorithms into natural science research has become increasingly feasible with the advancement in QML and quantum processors, enabling novel scientific research. By employing interpretable (or explainable) AI, it is possible to obtain insights into the decision‐making processes of AI, which is considered a “black box.” [457, 463] Well‐trained interpretable DNNs for estimating biochemical interactions may enable the inverse estimation of unknown in vivo parameters that affect nanostructure formation.

## 5 Conclusion and outlook

The current understanding of the structural development of three biomaterials, cellulose, chitin, and CBPs, was reviewed, highlighting similarities that may influence their tendency to form nanostructures. In contrast, melanin in melanosomes follows a distinct developmental pathway that requires co‐localization with other materials, including CBPs. The analysis was further extended to representative species containing these biomaterials, encompassing nanostructure formation from the molecular to tissue levels. A comprehensive overview of nanostructure formation is presented from biological, physical, and computational perspectives. Studies elucidating the complex dynamics and developmental pathways characterize the general mechanisms and key factors underlying biological photonic structures.

This review integrates biological, physical, and computational perspectives; however, several limitations should be carefully considered.

First, the discussion focuses on four representative biomaterials, cellulose, chitin, keratin, and melanin. Although these materials are widely distributed in nature, they do not encompass the full diversity of biological photonic systems. For instance, guanine‐based reflective structures found in fish [464], or organic–inorganic composite materials such as nacre [465], fall outside the present scope and may operate through distinct formation principles. Therefore, the hierarchical framework proposed in this review may not fully account for all possible pathways of biological nanostructure formation.

Second, the current understanding is largely based on structural characterization at different hierarchical levels, while comprehensive analysis of the entire developmental sequence remains technically challenging. Direct observation of intermediate states during in vivo formation continues to be difficult, and consequently, several formation mechanisms discussed herein are inferred from indirect evidence or reconstructed from mature end‐state structures. This limitation constrains the ability to definitively establish causal relationships between molecular‐level interactions and macroscopic architecture.

Third, computational approaches, including MD, DFT, and continuum simulation, inevitably rely on simplified assumptions regarding boundary conditions, environmental fluctuations, and biochemical heterogeneity. While these models provide valuable mechanistic insights, they do not fully capture the stochastic and multicomponent nature of biological self‐assembly. Accordingly, caution is required when extrapolating model predictions to in vivo systems.

Based on these insights, several important areas of research need to be explored in order to move toward a better understanding of how biological nanostructures form. First, the establishment of integrated multiscale modeling frameworks is essential. Future research should develop unified models that bridge molecular‐level chemical interactions, mesoscale organization, and macroscopic structure formation. These models must systematically relate intermolecular forces and thermodynamic considerations with the emergent optical functionalities arising from final structures. Importantly, these frameworks should incorporate time‐dependent evolution and environmental interactions to more faithfully reproduce in vivo assembly processes.

Second, comparative developmental studies across diverse taxa are necessary to distinguish conserved formation principles from lineage‐specific evolutionary adaptations. Expanding systematic comparative studies beyond a limited number of model organisms will help clarify whether strategies such as helicoidal organization, phase separation, templating, and spatial constraints are based on universal physical constraints or represent evolutionarily specialized solutions. In particular, contrasting polysaccharide‐based systems with melanin‐associated architectures may provide insight into how fundamentally distinct material properties converge toward similar optical outcomes.

Third, translating biological assembly principles into sustainable nanomanufacturing strategies represents a significant interdisciplinary opportunity. Rather than merely replicating final morphologies, future biomimetic fabrication should emulate the formation process itself. Nature constructs complex hierarchical structures from relatively limited sets of components under mild or ambient conditions, often relying on self‐organization and controlled growth across multiple length scales [466]. Leveraging these strategies may enable the realization of functional nanostructures via environmentally sustainable and economically viable fabrication pathways.

Future studies should move from reconstructing developmental processes from mature structures toward directly tracking nanostructure formation over time and testing proposed assembly mechanisms through controlled biological or physicochemical perturbations. Recent studies on other biological photonic systems illustrate that such an approach is becoming experimentally feasible and could be extended to the materials reviewed here. Developmental super‐resolution microscopy combined with actin perturbation has directly linked cytoskeletal organization to photonic ridge formation in butterfly scales [111], while multimodal imaging and pharmacological perturbations have resolved the cellular mechanisms underlying dynamic structural‐color changes in zebrafish iridophores [467]. More recently, live imaging and cryoFIB‐SEM combined with genetic and computational analyses have connected molecular interactions to the developing morphology of biogenic crystals [468].

A central long‐term goal should therefore be to establish quantitative relationships from biological regulation and molecular interactions to mesoscale organization, photonic architecture, and ultimately optical function, and to test these relationships prospectively by predicting how controlled perturbations alter the resulting structure and optical response. Achieving this level of mechanistic control would also create a more direct route from understanding natural assembly to engineering functional materials.

This translation is already emerging across several bioinspired material systems, including self‐assembled hydroxypropyl cellulose for responsive biomedical materials [290], chitin‐based optical films for colorimetric sensing [469], solid‐state polymerization‐induced phase separation methods that mimic the arrested phase‐separation process proposed for CBP‐based photonic structures in feather barbs [470], and melanin‐inspired nanoparticle assemblies that combine structural coloration with photothermal functionality [471]. Related optical design principles have also been implemented in spray‐coated Mie resonant paints for durable, highly non‐iridescent coloration [472], and deep‐foam photolithography that mimics natural amorphous structural whites through controlled pore formation and collapse to achieve high‐resolution optical patterning [473].

## Author Contributions

Conceptualization: S. K., J. K., S. M., J.‐S.Y. Supervision: J.‐S.Y. Funding acquisition: J.‐S.Y. Writing – original draft: S. K., J. K., S. M., J.‐S.Y. Writing – review & editing: S. K., J. K., S. M., L.D., M.D.S., J.‐S.Y.

## Funding

This work was supported by the National Research Foundation of Korea (NRF) grant funded by the Korea government (MSIT) (No. 2023R1A2C2006811), the BK21 FOUR (Fostering Outstanding Universities for Research) funded by the Ministry of Education (MOE) of Korea and National Research Foundation (NRF) of Korea (2025S1A5C3A05025824), the Institute of Convergence Science (ICONS), Center for JPIC, Yonsei University, and Human Frontier Science Program through the grant number (RGP0047/2019).

## Conflicts of Interest

The authors declare no conflicts of interest.

## Data Availability

Data sharing not applicable to this article as no datasets were generated or analysed during the current study.
